# Interpretable machine learning uncovers epithelial transcriptional rewiring and a role for Gelsolin in COPD

**DOI:** 10.1172/jci.insight.180239

**Published:** 2024-11-08

**Authors:** Justin Sui, Hanxi Xiao, Ugonna Mbaekwe, Nai-Chun Ting, Kaley Murday, Qianjiang Hu, Alyssa D. Gregory, Theodore S. Kapellos, Ali Öender Yildirim, Melanie Königshoff, Yingze Zhang, Frank Sciurba, Jishnu Das, Corrine R. Kliment

**Affiliations:** 1 Division of Pulmonary, Allergy and Critical Care Medicine,; 2 Department of Cellular and Molecular Pathology, and; 3 Center for Systems Immunology, Departments of Immunology and Computational & Systems Biology, University of Pittsburgh School of Medicine, Pittsburgh, Pennsylvania, USA.; 4 Institute of Lung Health and Immunity, Comprehensive Pneumology Center (CPC), Helmholtz Zentrum München, Member of the German Center for Lung Research (DZL), Munich, Germany.; 5 Institute of Experimental Pneumology, University Hospital, Ludwig Maximilians University (LMU) of Munich, Munich, Germany.; 6 Geriatric Research Education and Clinical Center (GRECC) at the VA Pittsburgh Healthcare System, Pittsburgh, Pennsylvania, USA.

**Keywords:** Cell biology, Pulmonology, COPD, Cytoskeleton

## Abstract

Transcriptomic analyses have advanced the understanding of complex disease pathophysiology including chronic obstructive pulmonary disease (COPD). However, identifying relevant biologic causative factors has been limited by the integration of high dimensionality data. COPD is characterized by lung destruction and inflammation, with smoke exposure being a major risk factor. To define previously unknown biological mechanisms in COPD, we utilized unsupervised and supervised interpretable machine learning analyses of single-cell RNA-Seq data from the mouse smoke-exposure model to identify significant latent factors (context-specific coexpression modules) impacting pathophysiology. The machine learning transcriptomic signatures coupled to protein networks uncovered a reduction in network complexity and new biological alterations in actin-associated gelsolin (GSN), which was transcriptionally linked to disease state. GSN was altered in airway epithelial cells in the mouse model and in human COPD. GSN was increased in plasma from patients with COPD, and smoke exposure resulted in enhanced GSN release from airway cells from patients with COPD. This method provides insights into rewiring of transcriptional networks that are associated with COPD pathogenesis and provides a translational analytical platform for other diseases.

## Abstract 2

We applied the novel interpretable machine learning models to the mouse model of COPD identify previously unrealized pathways earlier in human COPD. We found that gelsolin, an actin-regulating factor, in released from airway epithelial cells after CS exposure and is increased in the plasma of patients with COPD.

## Graphical abstract

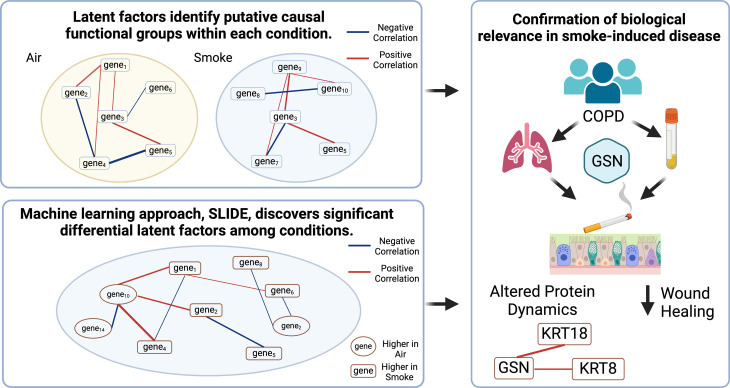

## Introduction

Chronic obstructive pulmonary disease (COPD) pathogenesis is characterized by alveolar destruction (emphysema), airway remodeling, and chronic inflammation. Notable changes occur in the epithelial, endothelial, and inflammatory cell lineages (1–5). Cigarette smoke (CS) results in the activation and alteration of a number of cellular function pathways in the host, such as immune activation, protease production, cytoskeletal remodeling, and cell death (6–9). Prior studies using standard methods of transcriptomic analysis of cell subpopulations in mouse and human lungs have informed our knowledge of COPD pathogenesis (1, 10). However, the understanding of cell-specific changes and transcriptional gene interactions in COPD remain limited due to the associated difficulties in achieving further granularity within high-dimensional data. The traditional single-cell RNA sequencing analysis pipeline provides differential gene expression however fails to identify gene interactions or latent factors that may be important for COPD pathogenesis.

To expand our understanding of the epithelial cell-specific transcriptional changes contributing to CS-induced lung injury, we utilized new unsupervised and supervised interpretable machine learning approaches to analyze single-cell RNA-Seq (scRNA-Seq) data from the mouse smoke exposure model. The CS exposure model in mice recapitulates many of the features of human disease, including chronic inflammation, cytoskeletal remodeling, cellular senescence, and alveolar epithelial cell injury (5, 11–16) and allows for the study of earlier disease processes. First, we used latent variable model with overlapping clusters (LOVE), an unsupervised latent factor approach that we coupled to the analyses of known protein-protein interactions to identify transcriptional signatures that were rewired by exposure to CS in the epithelial cell population. Next, we used significant latent factor interaction discovery and exploration (SLIDE) (17), a supervised interpretable latent factor regression approach on the expression data to uncover significant latent factors that can discriminate between epithelial cells exposed to air versus CS and provide insights into pathophysiological processes underlying the transcriptional rewiring. Using these interpretable machine learning approaches, we identified CS-induced transcriptional rewiring of cytoskeletal-related genes including gelsolin (GSN).

GSN is an actin-modifying cytoskeletal protein that has been shown to be increased in plasma from healthy smokers compared with healthy nonsmokers (18) and in the urine of individuals with α-1 antitrypsin deficiency–related COPD (19), and dysregulation has been implicated in other pulmonary diseases, including interstitial lung disease and acute lung injury (20–22). The role of GSN in CS-induced COPD remains unknown. Using the LOVE/SLIDE interpretable machine learning approach, we identified previously unknown epithelial cell–specific transcriptional rewiring of the actin-modifying gene *GSN* in CS-related lung disease and demonstrate its function in epithelial cells in COPD.

## Results

**Figure 1:**
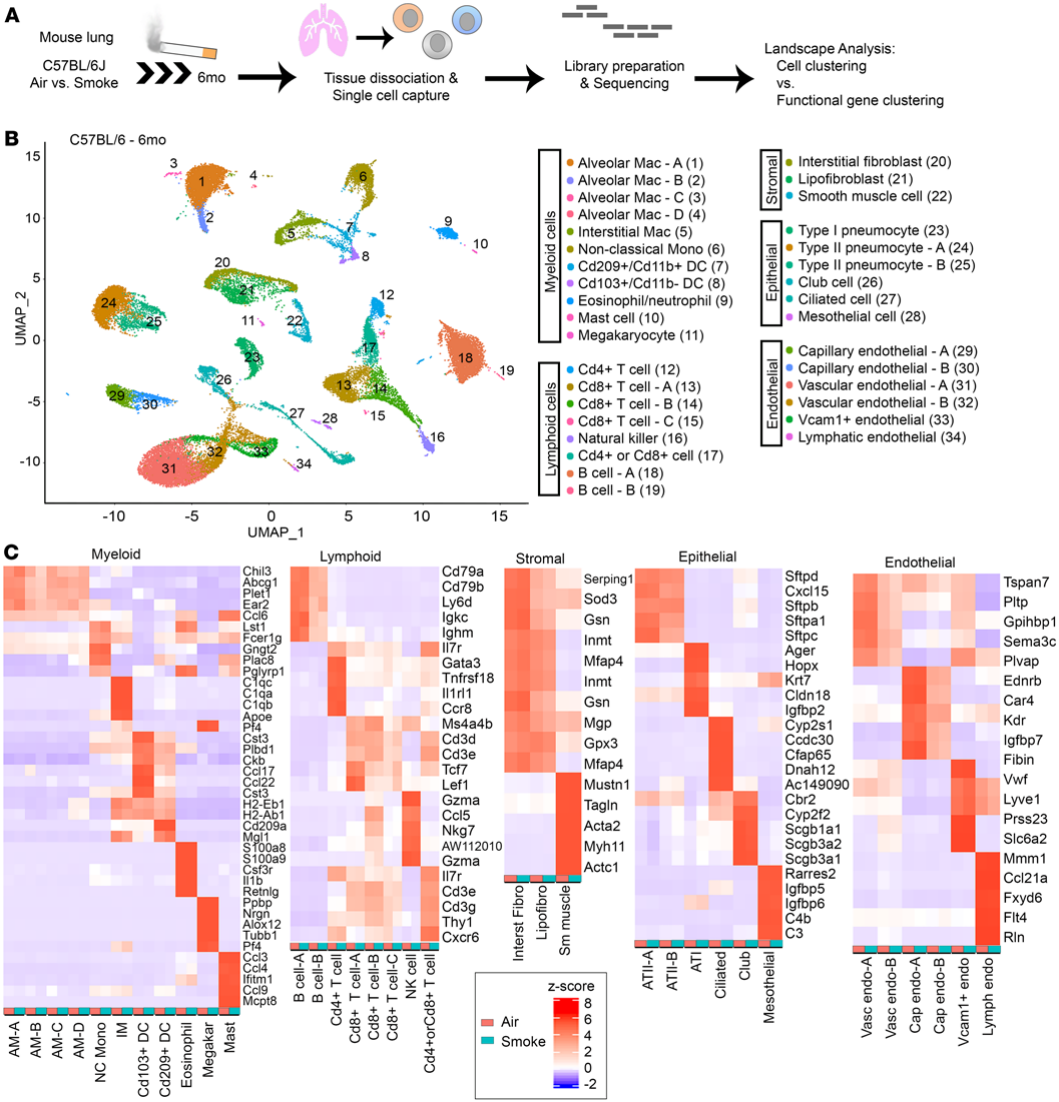
scRNA-Seq defines cell type profiles in a mouse model of COPD. C57BL/6J mice (*n* = 3 per group; 5,000 cells per mouse) were exposed to 6 months of air or CS. Harvested lung tissue was dissociated into single cell suspensions and processed individually for scRNA-Seq as described. (**A**) Experimental workflow and data analysis using standard Seurat cell clustering versus LOVE functional gene clustering. (**B**) Uniform Manifold Approximation and Projection (UMAP) representation of Seurat cell clustering identifying 34 cell clusters among the air and smoke exposed groups, notated with color and number labels (*n* = 4 individually sequenced mice per group). (**C**) Canonical cell marker genes identify distinct cell populations within myeloid, lymphoid, stromal, epithelial, and endothelial cell clusters. Groups are separated by exposure groups: air (red) or smoke (teal), *n* = 3 mice per group. Gene expression is reported as z-score by color histogram.

We analyzed scRNA-Seq profiles of single cell lung suspensions from C57BL/6J mice exposed to 6 months of air or CS using standard cell-centric analysis and a functional network-centric analysis called LOVE (Figure 1A). This standard smoke exposure model resulted in alveolar enlargement (emphysema) reflected as alveolar mean cord length, which we confirmed using 2 different analysis platforms (Supplemental Figure 1, A–D; supplemental material available online with this article; https://doi.org/10.1172/jci.insight.180239DS1). After 6 months of CS exposure, the final dataset consisted of analysis of 39,633 total cells, an average of 4,954 cells per mouse (*n* = 4 mice per group; Total cells: 20,578 air; 19,055 smoke). We identified 34 clusters representing 22 distinct cell types in both control and CS-exposed lungs based on representative marker genes, demonstrating inclusion of all known major cell lineages: myeloid, lymphoid, epithelial, endothelial, and stromal (Figure 1B). For cell type identification of the derived clusters, we compared previously defined gene expression lists for mouse lungs to the differential expressed genes representing each cluster (23). Cell clusters were found to express canonical markers for the respective cell types (Figure 1C). The *Scgb1a1* gene was detected in all cells in our analysis with increased expression levels in club cells, similar to that reported previously (23). We did not detect a distinct cluster of goblet cells, which were included in the club cell cluster based on *Bpifb1* gene expression. We observed only subtle changes in the majority of cell marker genes with CS exposure (Figure 1C, red versus teal columns by cell type).

Cell set frequencies by cluster were determined to examine the effect of CS on the lung cell distributions. Cell counts were determined by the number of cells identified per cluster and proportion was determined based on the total number of cells assayed per mouse. Exposure to 6 months of CS resulted in significant changes in cell set frequencies in immune cell subsets, with an increase in alveolar macrophage (AM) clusters A (AM^A^) and B (AM^B^), and a decrease in CD4^+^ T cells (Supplemental Figure 2A). There was also a decreasing trend in frequency of type II pneumocytes, cluster A (Supplemental Figure 2B). Shifts in the transcriptional state of cell clusters was subtle in most clusters using UMAP projection (Supplemental Figure 2C). We next determined the transcriptional rewiring that occurs due to CS using interpretable machine learning and network approaches on single-cell transcriptomic data from epithelial cell subsets. We define transcriptional rewiring as changes in transcriptional state due to an external stimulus, such as CS.

### Lung epithelial cell heterogeneity occurs after smoke exposure.

**Figure 2:**
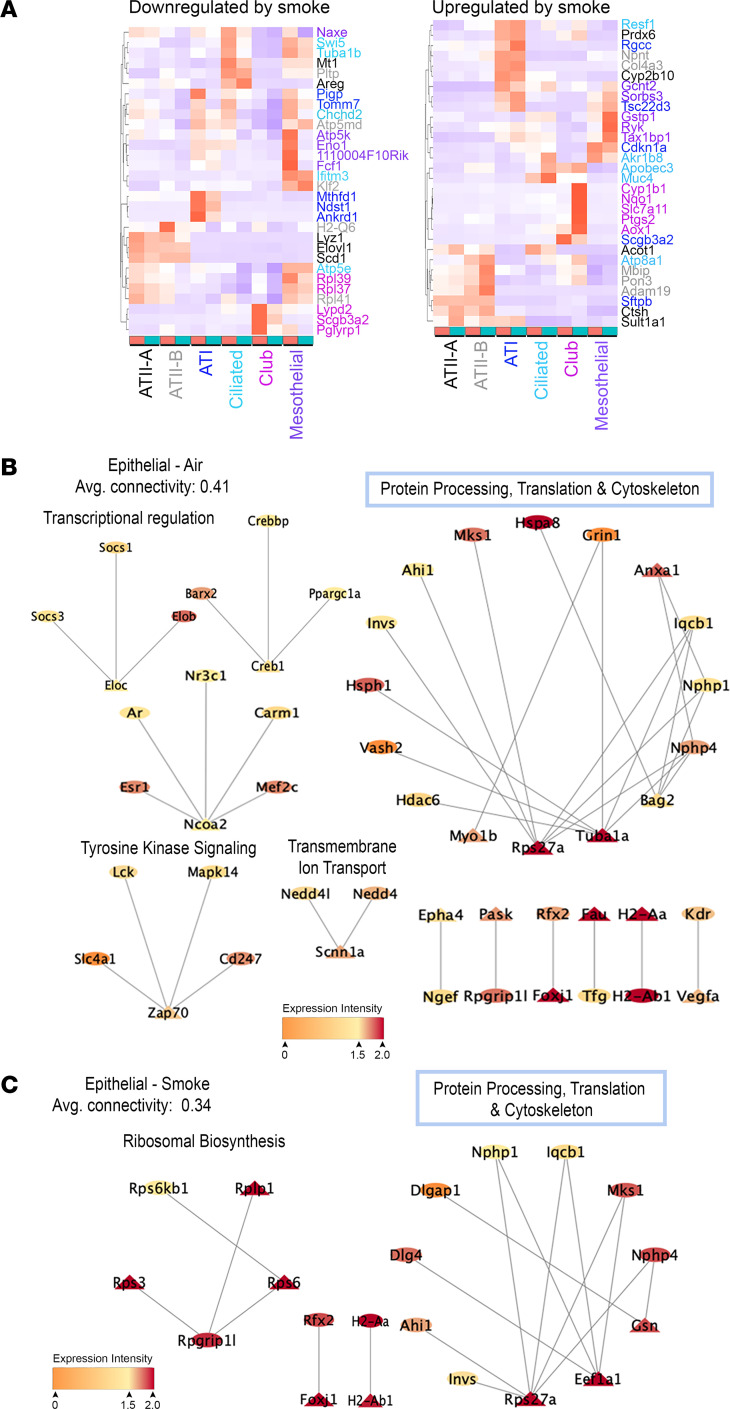
CS results in transcriptional rewiring within epithelial cells in the mouse lung. (**A**) Heat maps of z-scores showing the top 5 unique downregulated and upregulated genes between air and smoke exposure groups for each epithelial cell cluster. Groups are separated by exposure groups: air (red) or smoke (teal). Cell-type text color corresponds to the top 5 genes in the same color on the Y-axis label. Gene expression is reported as z-score by color histogram. (**B** and **C**) Epithelial cells were analyzed using a functional gene clustering model (LOVE) with functional gene clusters created for each exposure group. (**B**) Epithelial cells with air exposure, (**C**) Epithelial cells with 6 months smoke exposure. Functional groups were identified within air and smoke exposure groups. Gene nodes are shown as triangles, and predicted, experimentally validated interactor genes are shown as circles. Red-yellow shading represents the average gene expression intensity across all of the cells in that group. *n* = 3 mice per group.

After 6 months of CS exposure, we identified 6 distinct epithelial subclusters including alveolar type 1 pneumocytes (AT1), alveolar type 2 pneumocytes (AT2^A^ and AT2^B^), ciliated, club, and mesothelial cells (Figure 1, B and C). There was a nonsignificant trend toward a decrease in the cell frequency of the AT2^A^ subcluster with CS (Supplemental Figure 2B, *P =* 0.17). Among epithelial cells, AT2^A^ cells had the highest number of DEGs, followed by club, ciliated, AT2^B^, and mesothelial cells (Supplemental Table 1). We compared the top 5 differentially expressed genes (DEGs) between 6 months of air and CS exposure (Figure 2A, Supplemental Table 2), separating out unique genes from those that were up or downregulated in multiple cell types (Supplemental Figure 3, A–C). In AT1 and AT2 cell clusters, genes that were commonly upregulated included *Scgb1a1* and *Scgb3a1* (secretoglobins), *Cyp2f2* (cytochrome p450 2f2), and *Selenbp1* (selenium binding protein 1) (Supplemental Figure 2A). Upregulation of *Cbr2* was also shared with other epithelial cell types and has been implicated in alveolar epithelial cell plasticity (24). For AT1 cells, the top 5 unique downregulated genes (Figure 2A) were involved in pathways for folate and glucosamine metabolism (*Mthfd1* and *Ndst1),* mitochondrial protein import (*Tomm7*), and signal transduction (*Ankrd1* and *Pigp*). The top upregulated unique genes due to CS for AT1 cells included genes regulating surfactant metabolism (*Sftpb* and *Scgb3a2*) and cell cycle, including *Cdkn1a* (encodes for p21^Cip1^) and *Rgcc* (a cell cycle regulator that is induced by p53), the latter indicating an increase in the cellular senescence program (Figure 2A).

In the AT2^A^ cluster, there was down regulation of the growth factor–related genes (*Areg*), *Mt-1* (metallothionein-1), *Lyz1* (lysozyme 1), and lipid and fatty acid biosynthesis (*Scd1* and *Elovl1*), and upregulation of genes involved in cellular detoxification and oxidative stress (*Cyp2b10* and *Prdx6),* fatty acid metabolism (*Acot1*), and protein modification (*Sult1a1* and *Ctsh*). AT2^B^ cells had a downregulation of mitochondrial subunits of the electron transport chain for complex 1 (*Ndufa1, Ndufa2, Ndufa3, Ndufa6, Ndufa7, Ndufb10, Ndufb11, Ndufc1, Ndufv3, Ndufs5,* and *Ndufs6*), complex III (*Urcqc1, Cox5a, Cox6c, Cox7b,* and *Cox7c*), and ATP synthase *(Atp5md, Atp5a1, Atp5d, Atp5o*. *Atp5e,* and *Atp5g1)*. There were also reductions in ribosomal subunits (*Rpl41*), *Klf2* (kruppel like factor 2), *Pltp* (phospholipid transfer protein), and *H2-Q6* (histocompatibility complex). The top 5 unique upregulated genes in AT2^B^ represent matrix proteinases and matrix components (*Adam19* and *Col4a3*), and *Pon3,* *Npnt,* and *Mbip*. Polymorphisms in Adam19 have been associated with COPD development (25). These findings suggest a reduction in metabolic and translational programs in AT2 cells with increased focus on matrix interactions and kinase signaling.

Ciliated airway epithelial cells (CC) (Figure 2A) show downregulation of *Tuba1b* (tubulin), *Ifitm3* (interferon-induced transmembrane protein 3), *Swi5* (homologous recombination transcription factor), and genes involved in mitochondrial function (*Atp5e* and *Chchd2*). Further analysis of smoke-induced changes in CC gene expression demonstrate upregulation of genes responsible for mucin production (*Muc4*), inflammatory responses to viral infection (*Apobec3*), *Atp8a1* (ATPase phospholipid transporting protein 8a1, involved in ion transport), *Akr1b8* (aldo-keto reductase family member), and *Resf1* (retroelement silencing factor 1). Upregulation of mucin genes has also been seen in human ciliated epithelial cells from patients with COPD (6, 26). Club cells showed upregulation of cellular detoxification and oxidoreductase activity (*Cyp1b1, Nqo1,* and *Aox1)* and down regulation of ribosomal components (*Rpl39* and *Rpl37*), *Scgb3a2*, *Lypd2* (Ly6/Plaur domain containing protein), and *Pglyrp1* (peptidoglycan recognition protein 1).

### Epithelial cell-specific functional rewiring due to CS.

Our initial analysis using a traditional transcriptomic pipeline identifies differentially expressed genes and associated pathways. However, while informative, the DEG analyses are univariate, and downstream pathway analyses can only identify enrichment of genes of interest in known biological processes. They do not allow the identification of previously uncharacterized cellular programs, at the transcriptional level, underlying CS-induced lung injury. To this end, we utilized a combination of unsupervised and supervised machine learning approaches to characterize context-specific transcriptomic signatures underlying CS-induced lung disease in epithelial cells. First, we used LOVE, an unsupervised latent factor approach developed in-house (27), to uncover clusters corresponding to different functional states in these cells. There are 2 fundamental differences in our approach compared with the wide range of clustering approaches used routinely on scRNA-Seq data. First, rather than directly clustering cells, our model formulation allows us to cluster genes into latent functional states, which generates a corresponding assignment of cells to these latent states. Second, other latent factor approaches that are either nonidentifiable (i.e., the factors are unstable and change based on initialization conditions) or make very limiting assumptions regarding the structure of the data or data-generating mechanisms to provide identifiability of the latent factors (e.g., orthogonality of the latent factors). Our model comes with rigorous statistical guarantees regarding identifiability of these latent states without making any of these limiting assumptions (27). Among all epithelial cells, LOVE identified 12 clusters from the air scRNA-Seq data (Figure 2B) and 4 clusters from the smoke scRNA-Seq data (Figure 2C). For each cluster, LOVE identified a set of key marker genes that are representative of the functional states of these clusters. We then utilized what we consider to be a high-quality murine protein interactome network (28) to identify subnetworks involving proteins encoded by these marker genes. Coupling protein network analyses to key marker genes identified by LOVE from transcriptomic data provides insights into differences in key cellular programs across smoke and air in the epithelial cell types.

Our unsupervised LOVE analyses demonstrate distinct functional rewiring within epithelial cells due to CS (Figure 2, B and C). The epithelial air functional network (air exposed, healthy control mice) was highlighted by protein processing and cytoskeletal regulation (*Rps27a*, *Myo1b*, *Tuba1a*, *Bag2*, and *Anxa1*), transcriptional regulation (*Eloc, Creb1, and Ncoa2*), transmembrane ion transport (*Scnn1a*), and tyrosine kinase signaling (*Zap70*) (Figure 2B). With CS exposure, there was a decrease in the network complexity and rewiring of genes controlling the protein processing network (*Gsn*, *Rps27a*, and *Eef1a1*) and emergence of ribosomal biosynthesis as a dominant functional cluster (Figure 2C). The identification of *Gsn* as a primary node in the smoke exposure network is unique in COPD pathogenesis and was not previously identified using standard DEG analysis.

### Epithelial cell–specific latent factors are predictive of CS exposure.

The prior unsupervised analyses focus on defining functional states specific to mice with and without CS exposure. We next utilized a supervised interpretable machine learning approach to elucidate latent factors (context-specific gene modules) that are necessary and sufficient to discriminate between mice with and without CS exposure. Specifically, we used SLIDE to infer corresponding significant latent factors that provide insights into transcriptomic profiles underlying CS-induced lung injury among epithelial cell subtypes in our model. SLIDE is a first-in-class interpretable machine learning technique for identifying significant interacting latent factors underlying outcomes of interest from high-dimensional omic datasets. SLIDE makes no assumptions regarding data-generating mechanisms, comes with theoretical guarantees regarding identifiability of the latent factors/corresponding inference, and has rigorous FDR control. SLIDE outperforms/performs at least as well as a wide range of state-of-the-art approaches, including other latent factor approaches in terms of prediction (29). More importantly, it provides biological inference beyond prediction that other methods do not afford. These unique properties of SLIDE help move beyond correlative biomarkers to transcriptomic signatures regulating key processes underlying CS-induced lung injury in COPD.

**Figure 3:**
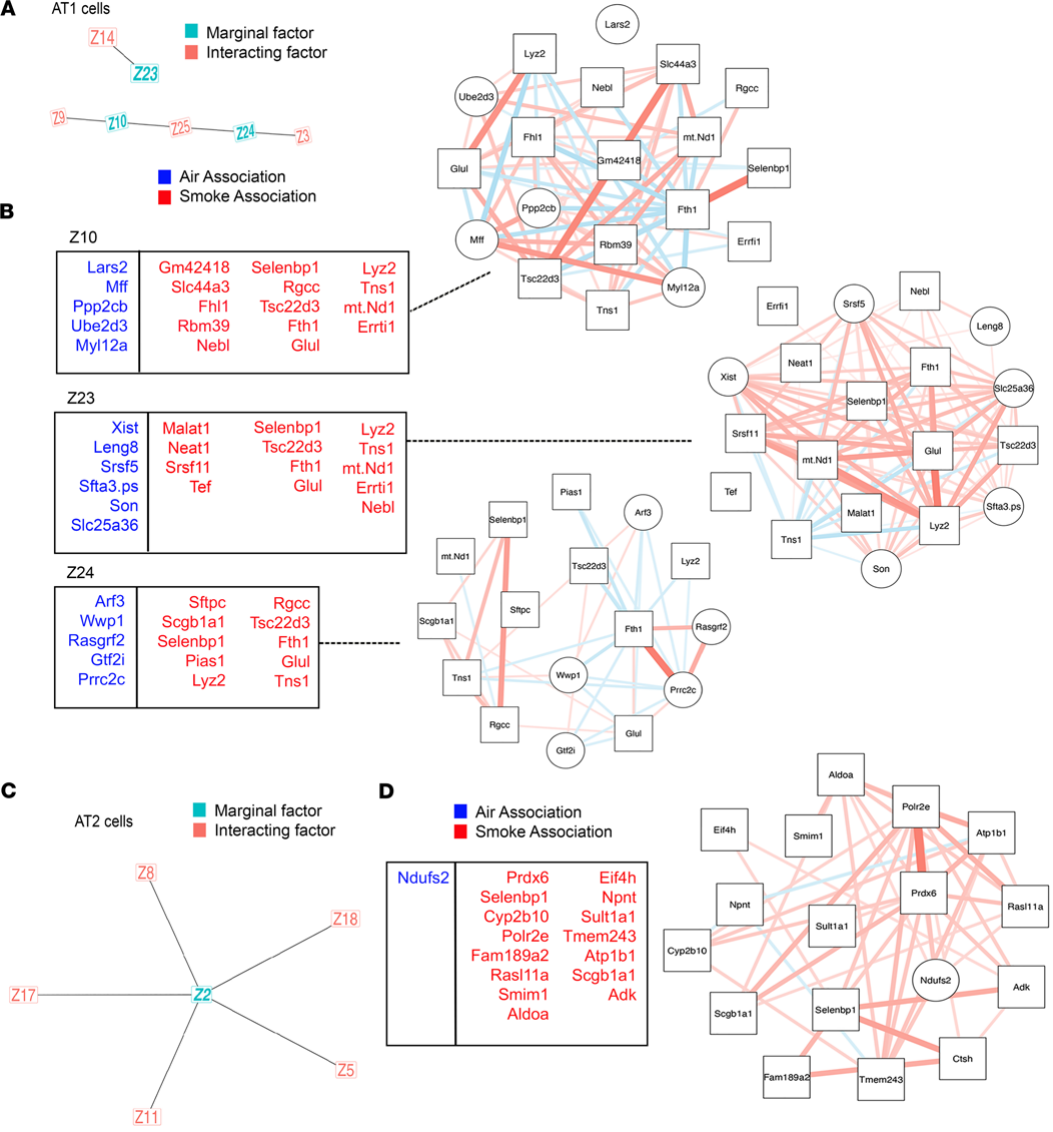
CS exposure resulted in transcriptional rewiring in AT1 and AT2 cell populations. scRNA-Seq data from mice exposed to 6 months of air or CS (*n* = 3 per group; 5,000 cells per mouse) were analyzed using SLIDE for dataset A. (**A**) Standalone significant latent (marginal) factors for AT1 cells are in teal and interacting latent factors are in red. Genes comprising each latent factor by cell type are reported in the table and network connectivity maps with genes that characterized CS exposure in red/squares and air in blue/circles. (**B**) Latent factor genes and network map for AT1 cells, (**C** and **D**) SLIDE latent, and interacting factors identified for AT2 cells with latent factor genes shown that characterize CS (red/squares) versus air (blue/circles).

**Figure 4:**
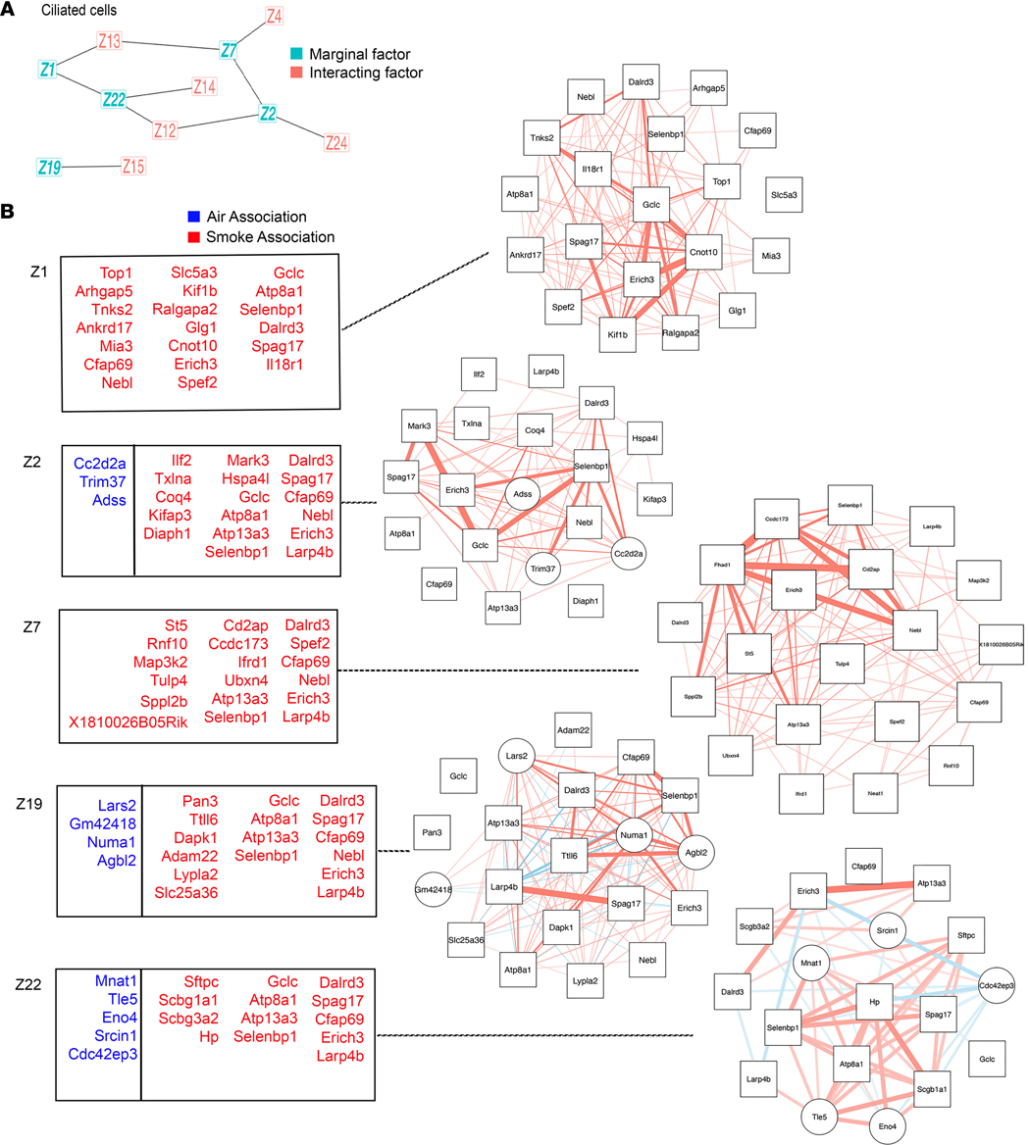
CS exposure resulted in transcriptional rewiring in ciliated cells. scRNA-Seq data from mice exposed to 6 months of air or CS (*n* = 3 per group; 5,000 cells per mouse) were analyzed using SLIDE for dataset A. (**A**) Standalone significant latent (marginal) factors for ciliated cells (CCs) are in teal and interacting latent factors are in red. (**B**) Genes comprising each latent factor for CCs are reported in the table and network connectivity maps with genes that characterized CS exposure in red/squares and air in blue/circles.

Using SLIDE, we performed analysis on AT2, AT1, and ciliated epithelial cell populations as identified by known cell markers within our scRNA-Seq dataset. Separate models were built for the different cell populations. Each of the models was able to meaningfully discriminate cells from mice with or without CS exposure (models were significant in a k-fold cross-validation framework with permutation testing). Within AT1 cells, 3 standalone significant latent factors were identified (significant stand-alone latent factor) with 4 interacting latent factors (Figure 3A). *Tsc22d3, Selenbp1,* and *Scgb1a1* were present in the standard DEG analysis and the SLIDE method. The significant stand-alone factors represent biological categories including cellular metabolism, the actin cytoskeleton and RNA regulation (Figure 3B). Within AT2 cells, we identified a stand-alone significant latent factor and 5 interacting latent factors (gene groups, Figure 3C). These factors included genes that were present in the standard DEG analysis and characterized in COPD in the literature: *Prdx6* (30, 31)*, Selenbp1* (32)*, Ctsh* (32, 33)*, Scgb1a1* (26), and *Ndufs2* (34) (Figure 3, C and D). However, SLIDE also uniquely identified genes that characterized CS exposure (*Polr2e, Fam189a2, Aldoa, Smim1, Rasl11a, Adk, Atp1b1, Tmem243, Sult1a1, Npnt,* and *Eif4a*) versus air exposure (*Ndufs2*) (Figure 3C). Interestingly, several of these genes would not be captured by traditional differential expression (DE) analysis as they are significant only in a multivariate setting (i.e., they are not significant by themselves but become significant in the context of context-specific gene groups). These CS-associated genes correspond broadly to antioxidant responses, drug metabolism, and transcriptional and protein regulation. For ciliated cells, an increased number of latent factors were identified compared with AT1 and AT2 cells. There were 5 standalone significant latent factors were identified with 6 interacting latent factors (Figure 4A). While a number of genes, *Sftpc, Scgb1a1, Atp8a1,* and *Selenbp1*, in the significant stand-alone latent factors were also captured by standard DE analyses, several genes were discovered by SLIDE for the first time. These CS-associated genes correspond broadly to cytoskeletal and vesicle regulation, antioxidant responses, drug metabolism, and RNA regulation (Figure 4B).

### Evaluation of the identified signatures in an orthogonal cohort.

**Figure 5:**
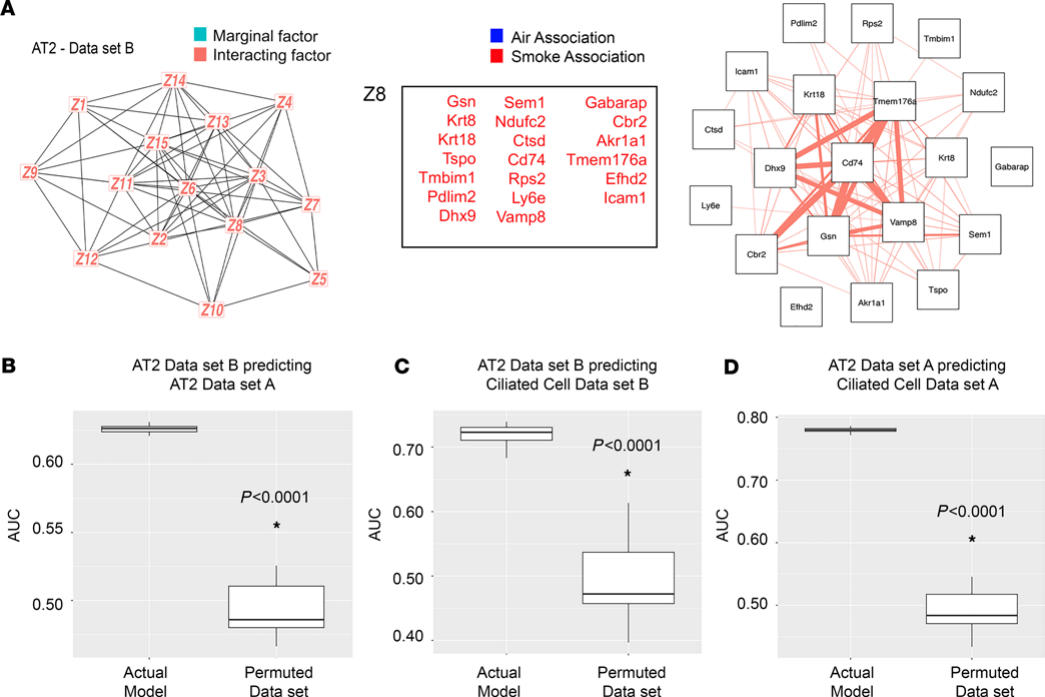
A *Gsn*-centric latent factor gene set can predict the smoke-exposed group within other epithelial cell types. scRNA-Seq data from mice exposed to 6 months of air or CS (*n* = 3 per group; 5,000 cells per mouse) were analyzed using SLIDE for dataset B for AT2 cells. (**A**) Standalone significant latent (marginal) factors for AT2 cells are in teal and interacting latent factors are in red. Genes comprising each latent factor by for the AT2 cells are reported in the table and network connectivity maps with genes that characterized CS exposure in red/squares and air in blue/circles. Of note, no air-associated latent factors (blue/circles) were present in this analysis. Cross prediction analysis was completed for between dataset A and B to determine if the CS treatment group could be identified. Area under the curve (AUC) is reported. Statistical comparison by 2-tailed Student’s *t* test with Mann-Whitney test for data in **B**–**D**. *P* values are noted. (**B**) AT2 dataset B predicting the CS group from AT2 cells in dataset A, (**C**) AT2 dataset B predicting the CS group from ciliated cells in dataset B, (**D**) AT2 dataset A predicting the CS group from ciliated cells in dataset A.

We then sought to further evaluate the robustness of the identified latent factors beyond the rigorous cross validation and permutation testing analyses outlined above by testing model performance on a completely orthogonal scRNA-Seq dataset (dataset B, GSE151674 as previously reported (15, 35)) in C57BL/6 mice exposed to 6 months of air versus CS performed by a separate research team. Specifically, we sought to assess the ability of SLIDE (17) to distinguish between cells with and without CS exposure. This is an extremely stringent evaluation as the model was built solely using our data, i.e., both the structure and prioritization of latent factors is using only our data. Indeed, the overall SLIDE model remained significantly predictive on dataset B, confirming the generalizability of our findings. Further, *Gsn* was identified in our LOVE model as a previously unknown and centric latent factor in lung epithelial cells in the context of CS (Figure 2C). Using our validation dataset B, *Gsn* was identified using SLIDE as a latent factor in the AT2 cells (Figure 5A). We next utilized the *Gsn*-centric latent factor gene set to determine if using this gene set can predict the air versus smoke-exposed groups within other epithelial cell types. Using the *Gsn*-centric gene list from AT2 cells (dataset B), we confirmed that SLIDE was able to predict the smoke-exposed group (Figure 5B) for AT2 cells in the original scRNA-Seq dataset (dataset A) and ciliated cells in dataset B (Figure 5C). In addition, the AT2 gene list from dataset A could similarly predict CS exposure across cell types, specifically ciliated cells from the same dataset (Figure 5D). This shows that network shifts due to CS exposure, which are represented by cell-type specific latent factors, can be used as predictive markers in other cell types exposed to CS.

### GSN is altered in human COPD.

**Figure 6:**
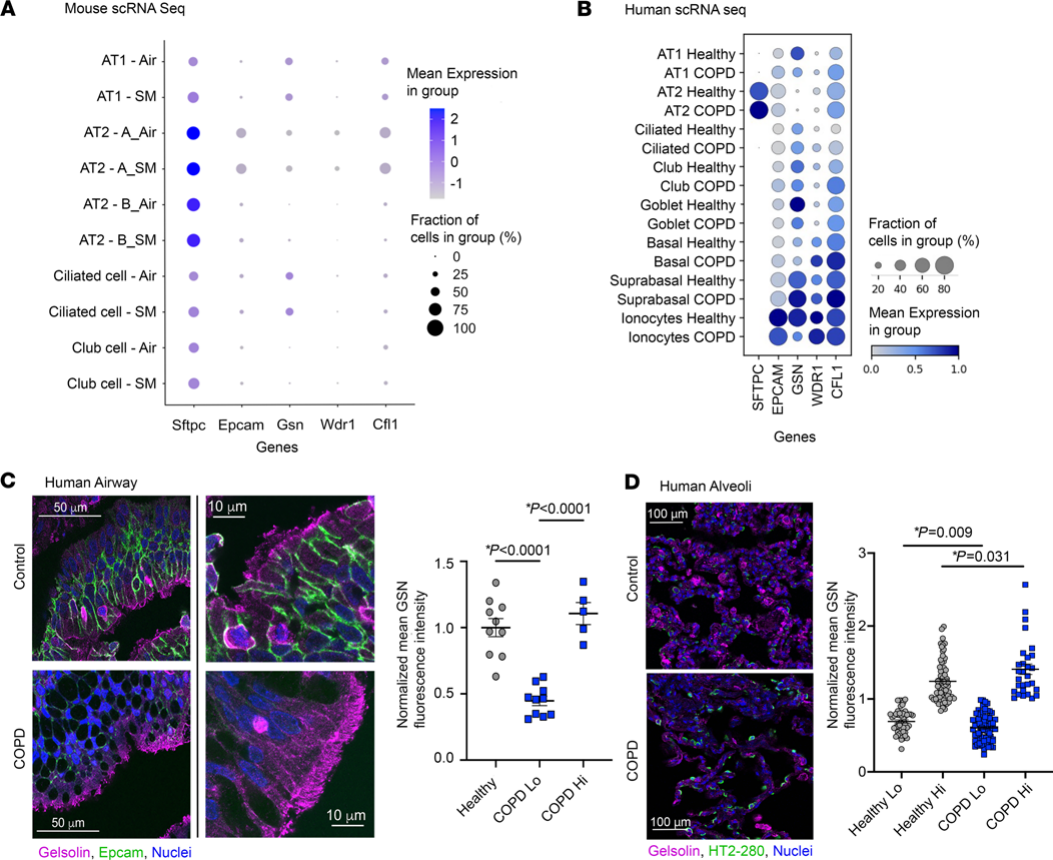
Gsn is enriched and increases in ciliated cells in mouse lung after CS and human COPD lung. Expression of *Gsn* was plotted from scRNA-Seq data from (**A**) Dataset A, epithelial cells isolated from mouse lung, 6 month smoke exposure model (*n* = 3 mice per group; 5,000 cells per mouse) and (**B**) human control versus COPD lung epithelial cells. Data are shown as mean gene expression in each group and fraction of cells with expression. Cells were isolated from lung samples from patients with COPD (GOLD stage IV, *n* = 6, ages 58–68, 5 males and 1 female) and normal nonsmoker donor controls (*n* = 4, ages 56–68, 3 males, 1 female). (**C** and **D**) Human lungs from people in the control group or patients with COPD was stained by IF and imaged on a confocal microscope *n* = 3–4 participants per group (8 images per participant). Data represent normalized mean grey value ± SEM. Statistically significant *P* values are noted. Statistics by 2-tailed Student’s *t* test with Mann-Whitney post test. Representative images are shown for (**C**) airway epithelium stained for GSN (magenta) and EPCAM (green). Scale bars: 50 μm (left) and 10 μm (right). (**D**) Alveolar epithelium stained for GSN (magenta) and HT2-280 (green). Scale bar: 100 μm. GSN staining intensity was quantified in HT2-280–negative, podoplanin-positive alveolar tissue. Mean grey intensities (per measured area) were normalized to the healthy control. Group data were split into high (Hi) and low (Lo) groups by using the mean value for the healthy control group.

We next validated a role for *Gsn* as a proof of concept that new biological targets can be identified using this computational analysis method. To determine which epithelial cells express GSN, we analyzed scRNA-Seq data from our mouse CS model and from healthy versus COPD lung. In the mouse lung, the percent of cells expressing *Gsn* is similar across cell types after CS exposure for 6 months (Figure 6A). In human lung, there was no statistically significant change in *GSN* expression in ciliated cells from COPD lungs (FC 0.135, *P* = 0.075), while there was a significant decrease in *GSN* expression level in AT1 cells (Figure 6B, log_2_FC –0.713, *P* = 1.07×10^–7^). *GSN* expression was very low in AT2 cells with no significant change between COPD and controls (log_2_FC –0.066, *P* = 0.190). This reduction in *GSN* in AT1 cells may reflect that the majority of the patients with COPD were not actively smoking, as lungs were procured at the time of lung transplant. Human lung tissue staining demonstrated that GSN is decreased in ciliated cells of the human COPD airway (Figure 6C) and confirmed that GSN is significantly reduced in the alveolar AT1 cells (podoplanin-positive cells) in COPD alveolar tissue (Figure 6D and Supplemental Figure 4).

**Figure 7:**
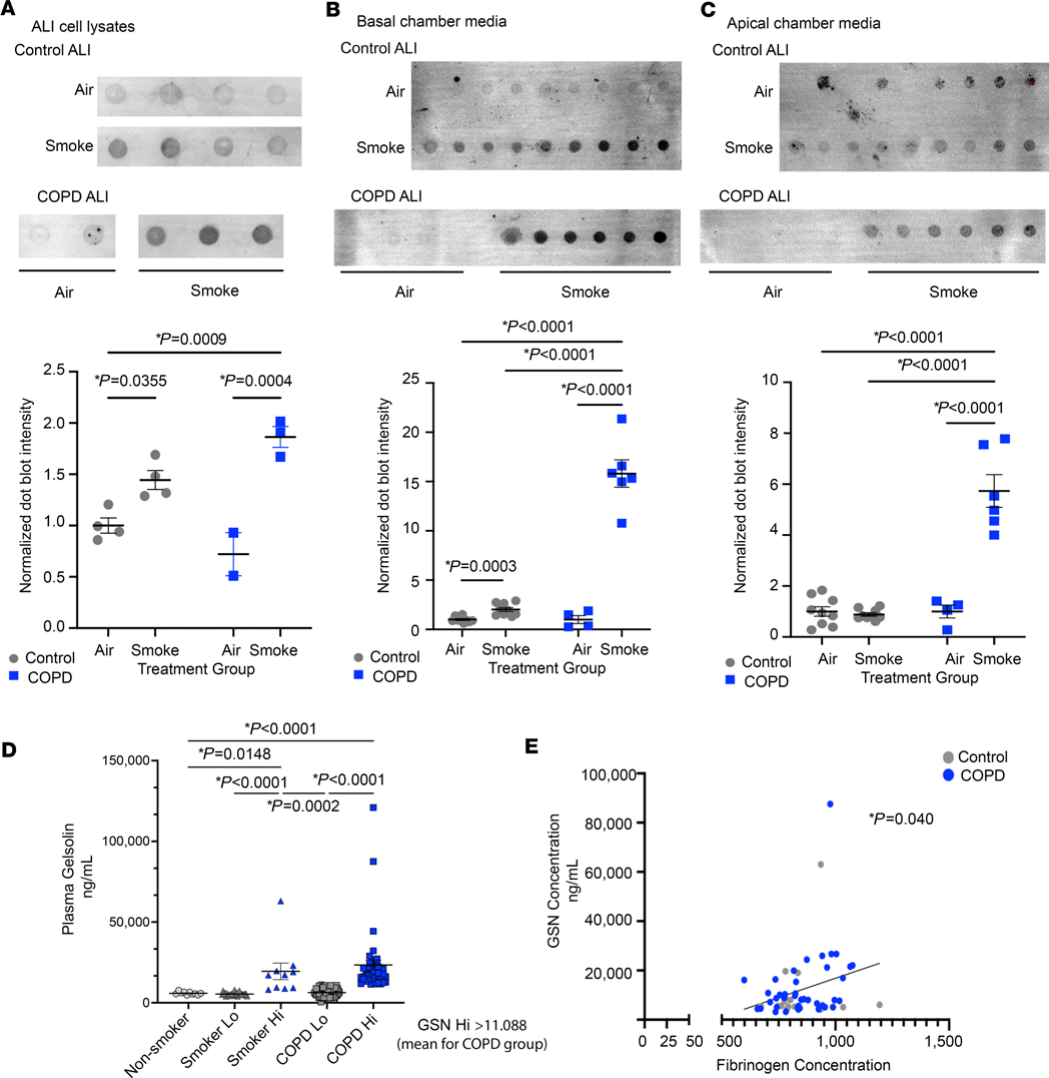
GSN is released by airway epithelial cells due to CS exposure and levels are increased in the plasma of patients with COPD. Primary human ALI cultures were exposed to gaseous CS by Vitrocell with *n* = 2 donors per group (biological replicates) and *n* = 2–9 inserts per donor (technical replicates). GSN quantity was determined by dot blot from (**A**) ALI cell lysates, (**B**) basal chamber media, and (**C**) apical chamber media. Data represent mean ± SEM. Statistically significant *P* values are noted. Statistics by 1-way ANOVA with Kruskal-Wallis post test. (**D**) GSN concentrations in human plasma were determined by ELISA for nonsmokers (*n* = 10), smokers without COPD (*n* = 38) and participants with COPD (*n* = 154). Data represent mean ± SEM. Statistically significant *P* values are noted. Statistics by 1-way ANOVA with Kruskal-Wallis post test. (**E**) Plasma GSN concentration compared with fibrinogen concentration in the same participant. Statistically significant *P* values are noted. Statistics by 1-way ANOVA with Kruskal-Wallis post test. Linear regression with *P* = 0.040. Grey, controls; Blue, participants with COPD.

GSN has been previously reported to be increased in the serum of patients with pulmonary fibrosis (21) and reduced in acute lung injury (22, 36). GSN can be released from cells as a plasma form (37). To determine if GSN is released from airway epithelial cells, we exposed primary human bronchial epithelial cells (HBECs) that were grown and differentiated to ciliated cells at air liquid interface (ALI) to control air or gaseous CS by Vitrocell exposure chambers (Vitrocell Inc). HBECs were attained from people in a normal control group and patients with COPD. We tested cell lysates, basal chamber media, and apical cell surface liquid for total GSN by dot blot analysis. There was an increase in GSN in cell lysates after CS exposure in normal HBECs with an exaggerated increase in COPD HBECs (Figure 7A). In normal HBECs, CS caused GSN to be released into the basal chamber media (Figure 7B). In COPD HBECs, GSN release was significantly higher into the basal chamber media and apical surface liquid compared with normal HBECs (Figure 7, B and C). These findings support that GSN expression and release is induced by CS in ciliated airway epithelial cells and further augmented in COPD. We next determined the level of GSN in human plasma from nonsmoking participants in the control group, active smokers without COPD, and patients with COPD by ELISA (Abcam Inc). GSN was significantly increased in the plasma of patients with COPD (*n* = 79) compared with controls (*n* = 10) and smokers without airflow obstruction (*n* = 38) (Figure 7D). There was no significant difference in GSN between participants in the control group and smokers. Plasma fibrinogen level has been previously reported as a biomarker of systemic inflammation in COPD (38, 39). GSN level correlated positively with plasma fibrinogen level in the same individual (Figure 7E, *P =* 0.040).

**Figure 8:**
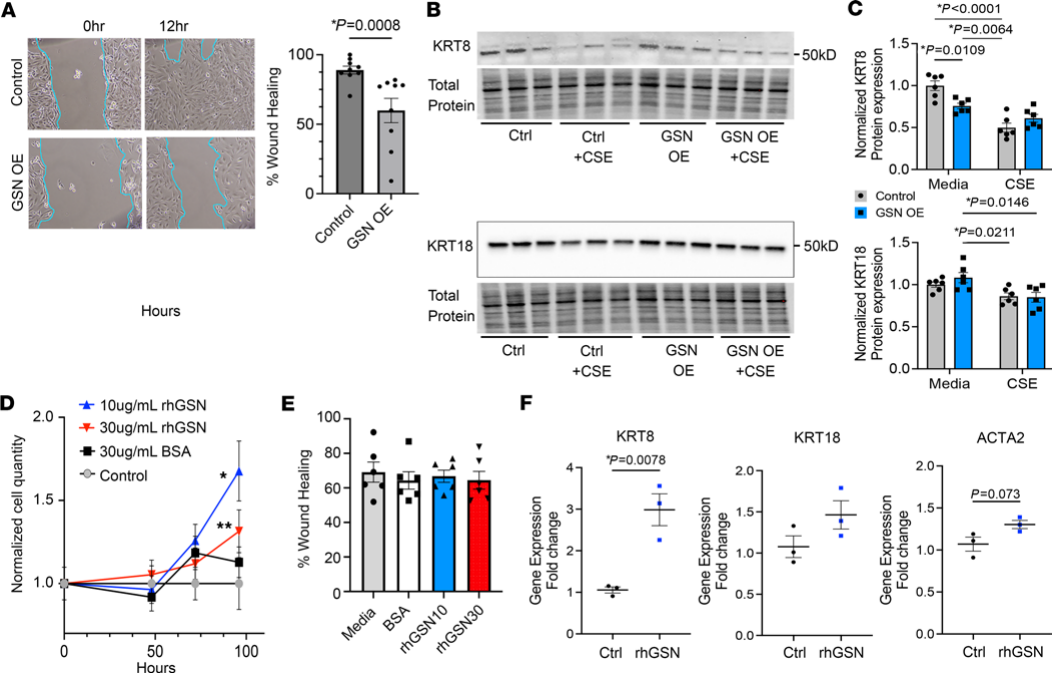
Endogenous overexpression of GSN and exogenous GSN alter cellular migration, proliferation, and KRT8 dynamics. Beas-2b cells were analyzed for wound healing, cellular proliferation, and cytokeratin expression in the context of GSN overexpression (GSN OE) or exogenous recombinant human GSN (rhGSN). (**A**) Wound healing assay with GSN OE. Wounds were measured at time 0 and 12 hours. Percent wound healing was calculated. Representative images are shown. Data represent *n* = 3–6 per group technical replicates and 2 separate experimental replicates. Statistical comparison by 2-tailed Student’s *t* test with Mann-Whitney test with *P* values noted. Images were acquired at 4X magnification. (**B**) Western blot for KRT8 and KRT18 was performed on Beas-2b cells with GSN OE compared with control, with or without 10% CSE exposure (24 hours). Data are representative of 2 separate experiments (*n* = 3 per group per experiment). (**C**) Quantification of Western blot band intensity for KRT8 and KRT18. Normalized data are representative of 2 separate experiments (*n* = 6 per group total). Statistics by 1-way ANOVA with Kruskal-Wallis post test with *P* values noted. (**D**) Cell proliferation was determined using the Cyquant assay in cells exposed to rhGSN at 10 or 30 μg/mL compared with media or BSA (30 μg/mL). Cells were assessed at 48, 72 and 96 hours. Simple linear regression analysis was performed. P values represent (*P = 0.0004, **P = 0.0145). (**E**) Wound healing assay was performed on Beas-2b cells treated with rhGSN at 10 or 30 μg/mL compared with cells treated with media or BSA (30 μg/ml). Wounds were measured at 0 and 12 hours. Percent wound healing was calculated. Representative images are shown. Data represent *n* = 6 wells per group. Statistical comparison by 1-way ANOVA with Kruskal-Wallis post test. (**F**) Beas-2b cells were exposed to rhGSN at 30 μg/mL for 24 hours with subsequent RT-PCR for *KRT8*, *KRT18,* and *ACTA2*. *n* = 3 biological replicates per group. Statistical comparison by a parametric 2-tailed Student’s *t* test.

We next determined the effect of GSN overexpression and exogenous GSN on epithelial cell migration and proliferation. In a 2D wound (scratch) assay using Beas-2b cells, GSN overexpression (OE) resulted in a decrease in wound healing over 12 hours (Figure 8A, *P =* 0.0008). GSN overexpression quantification is shown in Supplemental Figure 5. SLIDE analysis demonstrated transcriptional connections between GSN, cytokeratin 8 (KRT8), and cytokeratin 18 (KRT18) (Figure 5A). We examined this interaction through overexpression of GSN in Beas-2b cells in the context of CS extract exposure (10% CSE). GSN OE resulted in a decrease in KRT8 with a significant interaction between GSN OE and CSE treatment (*P =* 0.009 by 2-way ANOVA) (Figure 8, B and C). KRT18 was significantly reduced by CSE exposure in GSN OE cells (*P =* 0.015) but not control cells (*P =* 0.22) (Figure 8, B and C). We found that exogenous recombinant human GSN (rhGSN) promoted Beas-2b cell proliferation at 10 and 30 μg/mL (*P = 0.0004, **P = 0.0145) (Figure 8D). In the wound assay, rhGSN did not alter wound healing over 12 hours in Beas-2b cells (Figure 8E). rhGSN increased the gene expression of KRT8 (*P =* 0.0078) with a trending but nonsignificant increase in KRT18 and ACTA2 (α-smooth muscle actin) (Figure 8F).

## Discussion

This study provides what we believe to be new functional evidence of epithelial cell type–specific transcriptional rewiring that occurs in the context of CS exposure and identified a role for GSN in CS-induced COPD. We utilized unsupervised and supervised interpretable machine learning approaches, LOVE and SLIDE, to identify notable latent genes that are associated with our outcome of interest: CS-induced emphysema in the mouse model. The CS mouse model is a foundational model for the study of CS-induced lung injury and emulates many of the features of human COPD including alveolar tissue destruction (emphysema) and cytoskeletal remodeling, airway hyperplasia, and chronic inflammation (4, 5, 13, 14)). Critically, SLIDE moves beyond biomarkers to transcriptomic latent factors underlying biological changes due to CS exposure, as demonstrated with GSN in the human airway epithelium. This study therefore demonstrates feasibility and application of interpretable machine learning approaches for murine scRNA-Seq datasets to identify otherwise unknown genes and pathways involved in human COPD.

Our network analyses coupled to functional cluster identification using LOVE demonstrate that CS exposure results in functional rewiring of homeostatic transcriptional profiles in epithelial cells. A key advantage of the network approach coupled to latent factor analysis is the identification of functional modules, rather than only cell states, that are perturbed by CS. The rewiring of the functional modules across epithelial cells suggests the interplay of rewired cell-intrinsic and cell-extrinsic signaling in CS exposure. We found a reduction in the network complexity with CS exposure. This supports that CS causes the biological network across and within epithelial cell types to lose complexity, resulting in further loss of adaptation potential, robustness, and resilience in the epithelial cell network. Future studies could investigate and confirm this network deterioration in other cell types.

Dysregulation of GSN at the protein level has been demonstrated in small studies of healthy smokers (18) and genetic α-1 antitrypsin deficiency–related COPD (19). However, this study is the first to demonstrate alterations of GSN at the transcriptional and protein levels in CS-induced lung disease and COPD in mice and humans. GSN may be part of a molecular phenotype or signature of actin cytoskeletal remodeling in response to cellular injury or damage. Alternatively, cellular release of GSN may be a protective response to cellular injury. GSN can act as an actin scavenger by binding free actin subunits, which may be released into the extracellular space and plasma after cell damage in disease states such as sepsis and acute respiratory distress syndrome (40, 41). Recombinant human plasma GSN is also protective against acute lung injury due to bacterial infection (42, 43). Intracellular GSN effects are likely different than secreted GSN. Our in vitro wound assay suggests that GSN OE inhibits cell migration likely related to altered actin dynamics, while exogenous recombinant human GSN does not. However, exogenous GSN does promote cell proliferation. SLIDE analysis identified new transcriptional connections between GSN, KRT8, and KRT18, which was further supported by in vitro expression changes in KRT8. These KRT8 expression changes differed between GSN OE and exogenous GSN exposure. Keratins are abundant intermediate cytoskeletal filaments that regulate a number of cellular functions including cell shape and proliferation. They have also been implicated in pathogenesis and as markers of unique transitional epithelial cell states in the lung (44, 45). Further studies in human COPD and related mouse models will be important to clarify the implications of GSN on epithelial cell function and GSN-keratin interactions.

A limitation of the study is that gene expression may not always reflect protein concentration and function, therefore, validation of gene and protein expression are required. Single cell isolation is dependent upon isolation protocols, to which some cells may be more sensitive or difficult to recover. Variability is also introduced when different sequencing platforms are utilized. However, our cross-prediction analysis supports that SLIDE has strong predictive power both within and between complex sequencing datasets.

In summary, this study provides a transcriptional assessment of the CS exposure animal model for COPD research that complements the emerging transcriptional profiles established for human COPD. After CS exposure, there is transcriptional rewiring of epithelial cell types with a reduction in network complexity that informs future mechanistic studies and potential therapeutic avenues in COPD. Furthermore, we also successfully applied both the unsupervised latent factor–based LOVE and SLIDE models to murine scRNA-Seq data, which revealed functional transcriptional rewiring of the cytoskeletal network and validated a biological role for GSN in human COPD. This model can be applied to other disease-based datasets for biological discovery.

## Methods

### Sex was considered as a variable.

Human samples included both males and females, which were distributed evenly between normal control and COPD groups when possible. Mouse smoke exposure models utilized female mice, which demonstrate increased sensitivity to the emphysema phenotype due to smoke exposure.

### Human lung tissue and plasma samples.

Human lung tissue samples were obtained from The Airway Cell and Tissue Core at the University of Pittsburgh (supported by P30 DK072506, NIDDK, and the CFF RDP to the University of Pittsburgh). Donor lung samples were obtained from the Center for Organ Recovery and Education (CORE) at the University of Pittsburgh. Donor lung samples originated from lungs deemed unsuitable for organ transplantation. All COPD samples were from lungs explanted from patients with COPD who had undergone lung transplantation under an approved protocol. Lung tissues were stored at –80 °C until use. Plasma samples and clinical data consisted of participants in the COPD Specialized Centers of Clinically Oriented Research (SCCOR) cohort at the University of Pittsburgh. SCCOR is a single center COPD cohort recruited between July 2008 and June 2010 to study of the molecular and cellular basis of COPD subphenotypes. All SCCOR participants are 40 years of age or older with a minimum of 10 pack-year tobacco history at enrollment. Plasma samples were analyzed from 79 SCCOR participants with COPD, defined by a postbronchodilator forced expiratory volume in 1 second to forced vital capacity ratio (FEV_1_/FVC) of < 0.70. For control groups, 10 SCCOR participants without evidence of airflow obstruction on spirometry (controls) and 38 participants with active smoking but no evidence of COPD were selected. All samples were deidentified prior to acquisition for experimental testing.

### Animal smoke exposure and sample collection.

Animals were housed according to standard housing criteria. C57BL/6J mice were obtained from Jackson Laboratories (female mice at 10–12 weeks of age, *n* = 3–4 per group) and subjected to the smoke of 4 unfiltered cigarettes per day (lot# 3R4F; University of Kentucky), 5 days a week for a duration of 6 months, using a smoking apparatus that delivers targeted CS to single mice isolated in individual chambers (7, 46). The controls in each group were exposed to room air alone. These mice were caged separately and housed in the same facility as their smoke-exposed counterparts. At the completion of each experiment, mice were euthanized by CO_2_ inhalation, the chest was opened, and the trachea was cannulated. For scRNA-Seq data comprising dataset A, the left lung lobe was tied off, extracted, and placed in complete medium (DMEM with 10% FBS and 1% HEPES, 1M) on ice. The remaining lung lobes were inflated with 10% buffered formalin at a constant pressure of 25 cm H_2_O for 10 minutes. The lungs were then ligated, excised, and fixed in formalin for 24 hours before washing in PBS, storing in 70% ethanol, and embedding in paraffin. Serial midsagittal sections were obtained for histological analysis.

All animal experiments for dataset B collection were performed in accordance with the Animal Care and Use Committee of Helmholtz Zentrum München. Animals were housed according to standard housing criteria. C57BL/6J mice (female mice at 8–12 weeks of age) were subjected to full-body smoke exposure for 50 minutes twice daily (lot# 3R4F; University of Kentucky), 5 days a week for a duration of 6 months. The controls in each group were exposed to room air alone. At the completion of each experiment, mice were euthanized and lungs were collected for single cell isolation as previously reported (15, 35).

### Mouse lung morphology analysis.

Mouse lungs that were inflation fixed as described were imaged with brightfield at 20× magnification. Images were masked to block out airways, blood vessels, and intraalveolar immune cells or debris. Using an ImageJ–based script we called “WaffleFry”, each masked image was overlaid with horizontal and vertical lines interspaced at 10-pixel intervals. Chord length measurements were automatically processed vertically and horizontally across this image grid. A chord length measurement was recorded when the subsequent pixel had tissue density (rather than airspace). Chord lengths of under 8 pixels were discarded to prevent capture of nonalveolar spaces. This analysis tool is similar to previously validated programs (7, 46). It has been uploaded to Github at https://github.com/ckliment/WaffleFry.git (Commit ID: 0097d2b1e5ca6bc15ae97f769030a0109bccf62c).

Images were also analyzed using a published deep learning algorithm called “Deepmasker” to measure alveolar spaces with exclusion of intraalveolar inflammatory cells and blood vessels, as previously described (47), with comparable results (Supplemental Figure 1).

### Mouse single-cell lung processing and scRNA-Seq.

2 sets of scRNA-Seq data were analyzed for this study: dataset A from the University of Pittsburgh and dataset B from the Institute of Lung Health and Immunity, Helmholtz Munich, Germany.

For dataset A, the left lung lobe from each mouse was further processed to isolate single cell suspensions. Lung tissue was first minced into small pieces and placed in 0.5 mL dispase solution (50 U/mL, Sigma-Aldrich) in a conical tube. The tissue was incubated at room temperature on a gentle rocker for 45 minutes. The tissue was then placed in a petri dish with 7 mL complete medium with 10 μL DNase-I solution (1 mg/mL, Sigma-Aldrich), teased apart, and placed on a rocker for 12 minutes to liberate adherent cells. Liberated cells were then passed through a 70 μm strainer into a conical tube with subsequent washing of the strainer with 5 mL of media. This process was repeated with a 40 μm strainer. Cells were spun down at 400*g* for 10 minutes at 4°C. Cells were resuspended in 6 mL of red blood cell lysis buffer, incubated for 5 minutes, and spun down. Cell pellets were resuspended in 0.5 mL PBS with 0.4% BSA (Thermo Fisher Scientific), filtered a final time through a 40 μm FloMi filter tip (Bel-Art), and kept on ice. Cells were counted and 5,000 cells were processed. Individual cells were barcoded with library preparation using 3primeV2 reagents by 10X Genomics Chromium System per the manufacturer’s protocol. Next generation RNA-Seq was performed on the libraries using an Illumina NextSeq-500 by the UPMC Genome Center (Pittsburgh, Pennsylvania, USA). Raw data were demultiplexed using Cell Ranger 5.0.1 and the mkfastq function and then aligned to 10X Genomics’ mouse reference genome mm10-2020-A using cellranger count. Data can be downloaded from the Gene Expression Omnibus database with the accession code GSE277533.

Dataset B was collected, processed, and published as previously reported (15, 35). C57BL6 mice were exposed to 6 months of air or whole-body CS (5 days per week, Cigarette lot# 3R4F; University of Kentucky). Drop-Seq technology and computational pipeline was used for the dataset, as previously reported. Raw sequencing output and count matrices after basic QC filtering can be downloaded from the Gene Expression Omnibus database with the accession code GSE151674.

### scRNA-Seq analysis and clustering.

Data from datasets A and B were analyzed using the R package Seurat V3.2.3 and R V4.0.2 (48–50). Cells were filtered for greater than 200 genes, fewer than 3,000 genes, and less than 25% mitochondrial genes. Data normalization was performed using the standard Seurat workflow followed by clustering and visualization using uniform manifold approximation and projection (UMAP). Marker genes for each cell type were identified using the Seurat FindAllMarkers function, and cluster cell identities were manually assigned using canonical cell markers and previously established gene marker lists (23). Differentially expressed genes comparing CS-treated and air control cells were identified using the FindMarkers function, an implementation of a nonparametric Wilcoxon rank sum statistical test. Genes with *P* value less than 0.05 and absolute lnFC greater than 0.1 were used as inputs for pathway enrichment analysis. Gene ontology analysis was performed using WebGestalt, which utilizes the Fisher Exact Test, identifying genes with a FDR less than 10% (51).

### LOVE and SLIDE models for latent factor analysis.

We first removed transcripts and cells exhibiting high sparsity in the transcriptomic count matrix from Seurat. Specifically, we discard transcripts with over 99.5% zeros and cells with more than 90% zeros across all datasets. LOVE, a 3-step algorithm, then identifies latent factors in an unsupervised way (27). The first step leverages the data covariance matrix to determine the structure and quantity of latent factors. The subsequent step identifies the gene members of each latent factor, which include both pure and mixed variables. The final step allocates weights to the mixed variables for each latent factor, informed by the findings of the first 2 steps.

LOVE was first employed on the complete dataset for each cell type. We then performed a nonoverlapping assignment of genes to latent factors according to the allocation matrix outputted by LOVE, which details the weight of each gene within each latent factor. Specifically, each gene was exclusively attributed to the latent factor where it has maximal weight. This definitive assignment process revealed 2 principal latent factors for all datasets: one predominantly composed of homeostasis and house-keeping genes, and another comprising genes with potential functional differentiation.

To refine the data resolution, we applied LOVE in a nested manner to the 2 distinct feature groups. Following the post-LOVE analysis and a repeat of the definitive assignment, we isolated the most significant 50 features in the allocation matrix from each latent factor, both from the house-keeping gene analysis and the differential functional gene analysis. To further mitigate noise, we calculated the median expression of each transcript using nonzero values and excluded those transcripts whose expression resides in the lowest quantile under both experimental conditions. For every cell type and experimental condition, we constructed a protein-protein interaction network based on the interactions among the chosen genes by using the “OR” logic. Networks were visualized through Cytoscape.

### SLIDE analysis.

The scRNA-Seq data underwent initial processing via the Seurat pipeline, as previously detailed. For each cell type, we began by applying sparsity filtering to remove cells and genes exhibiting high levels of sparsity. Subsequently, for each distinct cell type, which included AT1, AT2, and ciliated cells, we performed the SLIDE analysis to identify cell type–specific latent factors that underlie air and smoke conditions. SLIDE is an interpretable machine learning approach designed to uncover significant interacting latent factors that underlie outcomes of interest within high-dimensional omic datasets (17). For each distinct cell type, which includes AT1, AT2, and ciliated cells, SLIDE is a 2-step process: the first involves a 10-fold cross-validation with 20 replicates to uncover latent factors (LOVE); the second step focuses on pinpointing significant independent and interactive latent factors with the iterative multistage knockoffs method. Spec (a frequency-based parameter to quantify the stability of the multi-stage knockoff approach) and iteration numbers were set as 0.2 and 300, respectively, for all cell types.

The top weighted gene members corresponding to each latent factor are presented in Figure 3. These members are a union of the top 10 weighted genes derived from the allocation matrix produced by SLIDE and the 10 highest correlated genes between the gene expression and the air/smoke condition. The correlation of each gene is quantified by calculating the AUC.

Correlation networks are visualized by employing the QGraph package in R. Vertices within the networks represent the top-weighted member in the corresponding latent factor. The intergene correlations are calculated using the Pearson correlation coefficient. To maintain clarity and relevance in the visualization, any correlation with a value below 0.1 is excluded from the plot.

### Human lung scRNA-Seq and analysis.

Lung samples from patients with COPD (GOLD stage IV, *n* = 6, ages 58–68, 5 males and 1 female) and normal nonsmoker donor controls (*n* = 4, ages 56–68, 3 males, 1 female) were enzymatically digested as previously described (52), enriched EpCAM^+^ lung cells of the distal lung were separated out, and scRNA-Seq was performed on the EpCAM^+^ lung cells. The sequencing results were analyzed using the Cell Ranger pipeline from 10x Genomics (v3.1.0, STAR v2.5.3a) and the Scanpy package (v1.8.0) (53). Reads were aligned to a hg38 human reference genome (GRCH38.97). Barcodes with less than 400 or more than 20,000 detected transcripts were excluded from the single cell RNA library. Cells with a high proportion of mitochondrial-encoded transcripts were excluded. Cells with high background mRNA contamination were detected using the R library package SoupX (54) and also excluded from analysis. Variable genes were selected and ranked, and 3,426 genes identified as occurring in at least 3 samples were used as input for principal component analysis. After preprocessing, cells were clustered using standard cell markers. AT2 cells were specifically identified using cells positive for surfactant proteins SFTPC and SFTPB, rather than Epcam, to improve cell identification. Differential gene expression was calculated following leiden cell clustering and batch alignment with BBKNN (55). UMAPs were generated in Seurat (50). UMAPs and dotplots showing gene expression were generated via scanpy’s pl.umap() and pl.dotplot() function, respectively. 

### GSN plasma analysis.

GSN quantification was completed on human plasma samples using a GSN ELISA (Abcam, cat# ab270215). Samples were diluted at 1:5,000 and analyzed according to the manufacturer’s instructions.

### Human ALI cultures and smoke exposure.

HBECs from normal and COPD lungs were attained from the University of Pittsburgh cell core as previously described (56, 57) with a protocol approved by the University of Pittsburgh Investigational Review Board. Samples were from *n* = 2 donors per group (biological replicates) and 2–9 inserts per donor (technical replicates). HBE cells were grown to 80%–90% confluence in collagen-coated flasks then seeded onto type I collagen-coated (50 μg/mL in 0.02 N acetic acid) transparent PET Transwell inserts (0.4 μm pore, 24-well insert size 0.33 cm^2^ at a density of approximately 5–6 × 10^5^ cells/cm^2^. Once a confluent monolayer had formed on the inserts, the apical medium was removed, and cells were grown at ALI (58) over 3–6 weeks for differentiation into ciliated airway epithelium prior to experimental use.

The Vitrocell smoke exposure chamber was used to expose HBEs grown at ALI to humidified air or CS. A single exposure is considered air for 16 minutes or 2 cigarettes over sixteen minutes using an ISO standard protocol. ALI cultures were exposed to 2 cigarettes followed by a 2-hour break in a cell culture incubator (5% CO_2_, 37°C) then 2 additional cigarettes. After the second exposure, 100 μL of sterile PBS containing 1 × Halt protease and phosphatase inhibitor cocktail (Thermo Fisher Scientific no. 1861281) was added to the apical cell surface and cells were incubated for 1 hour prior to harvest of apical liquid, basal media, and cell lysates. Protease inhibitor cocktail was added to the basal media prior to collection. Cell lysates were collected using 1 × RIPA lysis buffer (Pierce no. 89900) supplemented with 1 × RNAase-free DNAase (Thermo Fisher Scientific no. EN0521) and 1 × Halt protease and phosphatase inhibitor cocktail and lysed by sonication. Samples were analyzed by dot blot analysis as described below.

### Tissue and cell immunofluorescent staining.

Immunofluorescent staining of lung tissue and cells in culture was completed as previously described (46). Lung tissue from human (normal control and COPD lungs) and mouse lung (air and CS exposed) were formalin fixed and paraffin embedded. Human lung tissue was analyzed from 3 patients in a control group (nonsmokers without lung disease) and 3 patients with COPD (GOLD stage 4). Prior to staining and analysis, tissue sections were deparaffinized and rehydrated by a series of xylene and ethanol washes. Antigen retrieval was completed using sodium citrate buffer, pH 6.0 at 95°C. Tissue was permeabilized with 0.3% Triton X-100 and 1% BSA in PBS and blocked with 2% BSA in PBS. Human and mouse lung sections were stained for mouse anti-GSN (1:100, Invitrogen no. 27752), rabbit anti-epcam (1:100, Abcam no. ab223582), HT2-280 (Terrace Biotech, no. TB-27AHT2-280), podoplanin (Developmental Studies Hybridoma Bank, Univ. of Iowa, Anti-Pdpn no. 8.1.1) and secondary Alexa fluorophore antibodies, including goat anti-mouse Alexa 555 (Invitrogen, cat. A32727), goat anti-mouse Alexa 647 (Invitrogen, cat. A21235), goat anti-rat Alexa 647 (Invitrogen, cat. A21247), and goat anti-rabbit Alexa 647 (Invitrogen, cat. A21245). Control sections were stained with nonimmune rabbit IgG (no. 2729P, Cell signaling). All tissue sections were stained with Hoechst at 10 mg/mL for 10 minutes. Sections were mounted with Prolong Gold (Molecular Probes, Thermo Fisher Scientific) and cured for at least 24 hours at 4°C prior to imaging. Images were acquired on a Nikon A1R confocal microscope using a 60 × objective.

After immunofluorescent staining, human lung sections (*n* = 3 healthy controls and *n* = 3 COPD, GOLD stage 4) were analyzed for *GSN* expression using ImageJ in airway epithelial cells or alveolar tissue. Images were acquired from different regions of the lung representing airways or alveolar tissue (*n* = 6–8 images each). Images were divided into 4 quadrants, and areas to be analyzed were sampled from HT2-280–negative stretches of podoplanin-positive alveolar tissue from each image quadrant and from the center of each image (5 areas per image). Macrophages and alveolar type 2 cells were avoided during analysis based on cell morphology and HT2-280 staining, respectively. Mean grey value for each area was recorded and compared between samples from individuals who were healthy and who had COPD.

### Dot blot and Western protein analysis.

For dot blot analysis, a PVDF Immobilon membrane (Bio-Rad no. 162-0177) was activated using methanol then washed in distilled, deionized water for 5 minutes 3 times. The membrane was layered with soaked blotting papers into the Dot Blot apparatus and 50 μL of each sample (apical surface liquid, basal media or cell lysates) were blotted onto a membrane in triplicate using gentle vacuum for 60 seconds. Blots were incubated in blocking solution (5% milk in PBS-tween) for 1 hour at room temperature followed by 3 rinses in PBS-Tween (PBS-T). The membrane was subsequently incubated with primary antibody for 1 hour at room temperature on a rocker, washed 5 times in PBS-T for 5 minutes, then incubated with secondary antibody for 1 hour at room temperature on a rocker followed by repeat wash steps. Western blot analysis was completed according to previously published methods (59). Primary antibodies used were targeted against GSN (1:500, Invitrogen no. 27752), DDK (1:1000, Invitrogen no. MA1-9187), KRT8 (1:500, DSHB no. TROMA-1) and KRT18 (1:500, Abcam no. ab93741). Secondary antibodies used were Invitrogen GOXMO HRP high XADS (no. A16078). Blots were developed using SuperSignal West Pico PLUS Chemiluminescent Substrate (Thermo Fisher Scientific no. 34580) and imaged using a ChemiDoc XRS+ (Bio-Rad) detector.

### GSN overexpression, exogenous GSN exposure, and wound healing assay.

BEAS-2B cells, a human bronchial epithelial cell line (ATCC), were grown and maintained in DMEM with Nutrient Mixture F-12 (Genesee Scientific) and supplemented with 5% FBS and 1% penicillin/streptomycin(Genesee Scientific). Cells were used between passage 3–8. Cells were cultured on tissue culture–treated polystyrene plates and were propagated when 70%–90% confluent. Cells were propagated by trypsinization with 0.25% trypsin-EDTA (Gibco no. 25200-056) and neutralized by trypsin neutralizing solution (Gibco no. R-002-100). For GSN OE, cells were transfected with 2.3 nM plasmid DNA for GSN (GSN_OHu20043D_pcDNA3.1+/C -(K)-DYK, Clone ID:OHu20043D, catalog no. SC1200, Genscript) or control (pcDNA3.1-C-(k)DYK, catalog no. SC1317, Genscript) using Lipofectamine 3000 (Invitrogen) per the manufacturer’s protocol. Select experiments were exposed to CS extract (CSE) as described below. Transgene expression was confirmed using Western blot analysis according to previously published methods (59) with GSN antibody (1:500, Invitrogen no. 27752) and DDK antibody (1:1000, Invitrogen no. MA1-9187). Analysis of KRT8 and KRT18 by Western blot was assessed using anti-KRT8 and anti-KRT18 antibodies above. Beas-2b cells were exposed to exogenous recombinant human GSN (rhGSN, Cytoskeleton Inc.) for 24–96 hours at concentrations of 10 or 30 μg/mL. Media alone or sterile BSA (at 30 μg/mL) were used as control conditions. Cells were analyzed for cell proliferation using the Cyquant assay, wound healing assay as described (analysis at time 0 and 12 hours), and real time PCR analysis at 24 hours for *KRT8*, *KRT18,* and *ACTA2*.

### CSE.

Research cigarettes were purchased from the University of Kentucky (Lot no. 1R6F). CSE was prepared in 30 mL of DMEM with 1% penicillin/streptomycin drawn into a 60 mL syringe (Thermo Fisher Scientific). A single cigarette was fixed on the top of the syringe and burned. The plunger was used to draw the smoke into the syringe followed by shaking to promote dissolution in the media. The CSE solution was filtered through a 0.45 μM filter. The absorbance of the CSE was measured using a cuvette at an O.D. of 310 nm with microplate reader (SpectraMax, M2, Molecular Devices). An O.D. value of 0.2 is approximately equal to 100%. The CSE was adjusted to 10% and used for cell exposure for 24 hours.

### Real-time PCR.

RNA was isolated from cells and converted to cDNA for real-time quantitative PCR (q-PCR) using LightCycler 480 SYBR Green I Master mix (Roche) and specific primer combinations on a CFX384 Real-Time system C1000 Touch Thermal Cycler (Bio-Red). Primers were purchased from Integrated DNA technologies, IDT including *ACTA2*: Hs.PT.56a.2542642; *KRT8*: Hs.PT.58.22681010; *KRT18*: Hs.PT.58.19252426 and *GAPDH*: Hs.PT.39a.22214836. Each sample using primers pairs was measured at least in duplicate. Relative fold change was calculated by normalizing to *GAPDH*.

### Statistics.

Mean densitometry (ImageLab Software) and all other quantitative data (mean ± SEM) were normalized to appropriate control groups. Statistical analyses were completed using GraphPad Prism 9.3. Data were assessed for sample distribution. If samples were normally distributed, then data are analyzed using ANOVA with Fisher’s LSD post test. If the data were not normally distributed, nonparametric analyses, including Kruskal-Wallis, Tukey’s, and/or Mann-Whitney were used. The association between circulating plasma GSN were assessed with linear regression analysis. For all statistics, a *P* value less than 0.05 was considered to be statistically significant.

### Study approval.

Human lung tissue samples were obtained from The Airway Cell and Tissue Core at the University of Pittsburgh. The tissue core and biorepository efforts are approved through the University of Pittsburgh Institutional Review Board (IRB) through the Human Research Protection Office (Pittsburgh, Pennsylvania, USA). The IRBs cover the procurement, processing, and distribution of human biospecimens (Total Transplant Care Protocol; IRB no. 2017H0309 and IRB PRO14010265) and the collection and distribution of clinical data (Honest Broker Protocol; IRB no. 2017H0310). Plasma samples and clinical data consisted of participants in the COPD Specialized Centers of Clinically Oriented Research (SCCOR) cohort, which is approved by the IRB at the University of Pittsburgh (IRB no. 19090239, Pittsburgh, Pennsylvania, USA). All animal experiments for dataset A collection were approved by and performed in accordance with the Institutional Animal Care and Use Committee (IACUC) of the University of Pittsburgh (Pittsburgh, Pennsylvania, USA).

### Data availability.

All data associated with this study are available in the main text or the supplemental materials including the Supporting Data Values File. scRNA-Seq data from C57BL/6 mice exposed to air or CS for 6 months is available from Geo Omnibus, GSE277533. Contact Corrine Kliment (ckliment@pitt.edu) for study correspondence and material requests.

## Author contributions

CRK, JD, and ADG conceptualized the project. JS, HX, CRK, YZ, QH, UM, NCT, and KM were responsible for computational analysis, experimental assays, and data processing. FS and YZ were responsible Human Cohort management (SCCOR). CRK and JD acquired funding. CRK, JD, HX, and JS wrote the original draft of the manuscript. CRK, JD, ADG, JS, TSK, AOY, and MK reviewed and edited the manuscript.

## Supplementary Material

Supplemental data

Unedited blot and gel images

Supplemental table 1

Supplemental table 2

Supporting data values

## Version 1 10/01/2024

In-Press Preview

## Version 2 11/08/2024

Electronic publication
