# Supplementary material for: Interpretable machine learning uncovers epithelial transcriptional rewiring and a role for Gelsolin in COPD
Source: JCI Insight. 2024 Nov 8;9(21):e180239. doi: 10.1172/jci.insight.180239 (PMC11601586; doi:10.1172/jci.insight.180239)
Supplement: Supplemental data [file jciinsight-9-180239-s255.pdf]

Supplementary Materials for

Interpretable machine learning uncovers epithelial transcriptional rewiring and a role for Gelsolin in COPD

Sui *et al.*

*Corresponding author. Email: ckliment@pitt.edu

This PDF file includes:

Figs. S1 to S5
Tables S1 to S2 (legends)

Other Supplementary Materials for this manuscript include the following:
Tables S1 to S2 files
Lung tissue morphology analysis program “WaffleFry” has been uploaded to GitHub

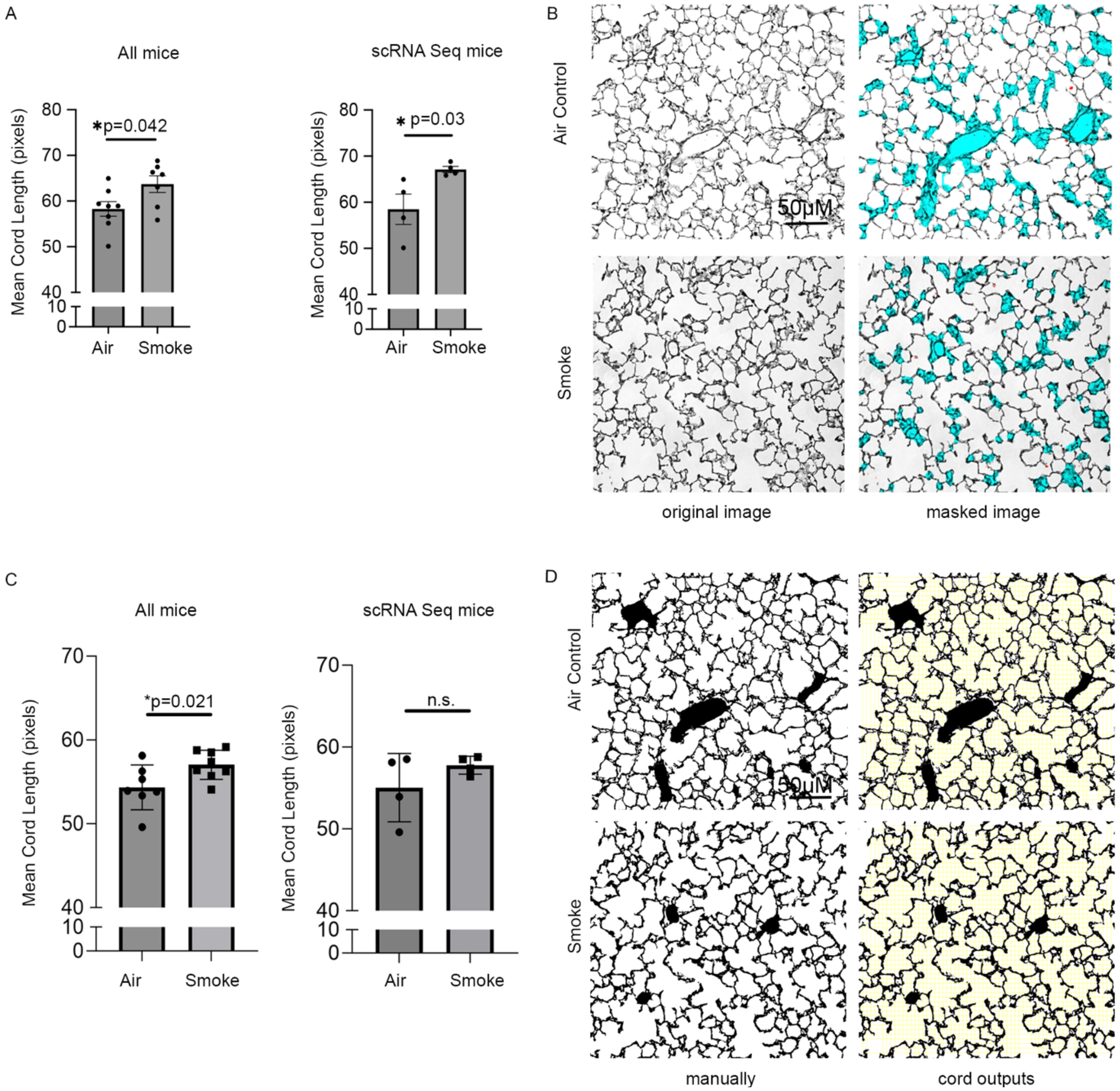

Figure S1. Mice exposed to cigarette smoke for 6 months develop alveolar enlargement. Mouse lung tissue was stained by H&E and imaged at 20X magnification. Images were analyzed for mean cord length using two analysis program. **(A)** Analysis using the DeepMasker program with machine learning masking and cord length measurement. Mean cord length is reported in pixels ± SEM. N=4-8 mice, 10-13 images per animal. Statistics by Student's t-test with Mann-Whitney post-test, *p-values are noted. **(B)** Representative images of grey scale images without (left) and with masking (right). Scale bar 50μm. **(C)** Analysis using ImageJ, conversion to grey scale, manual masking and the WaffleFry program. Mean cord length is reported in pixels. N=4-8 mice, 10-13 images per animal. Statistics by Student's t-test, *p-values are noted. **(D)** Representative images of grey scale images with masking only (left) and with masking and analysis lines in yellow (right). Scale bar 50μm.

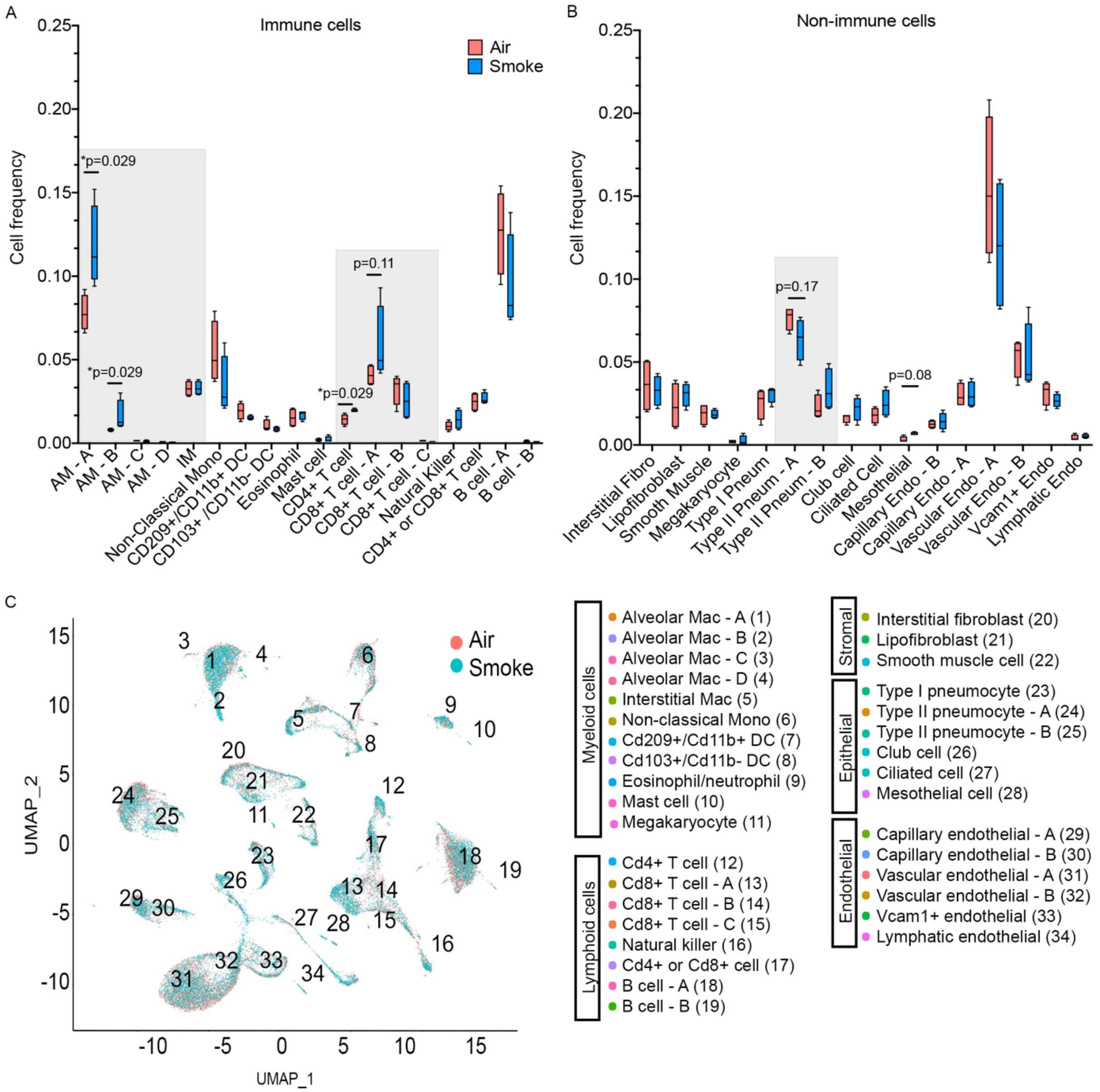

Figure S2 (A) Cell frequencies were determined for the epithelial sub-clusters (# of analyzed cells by cell type / total cells) for air and CS groups (n = 4 mice per group, 5,000 cells per mouse). Mean with standard deviation is shown. Statistical analysis using ANOVA with Mann-Whitney test, p-values are noted. **(B)** UMAP clustering of mouse lung cells displayed by exposure (air – red; smoke – teal). Cell clusters are labeled according to the corresponding numbers in figure 1B. (n = 4 mice per group, 5,000 cells per mouse).

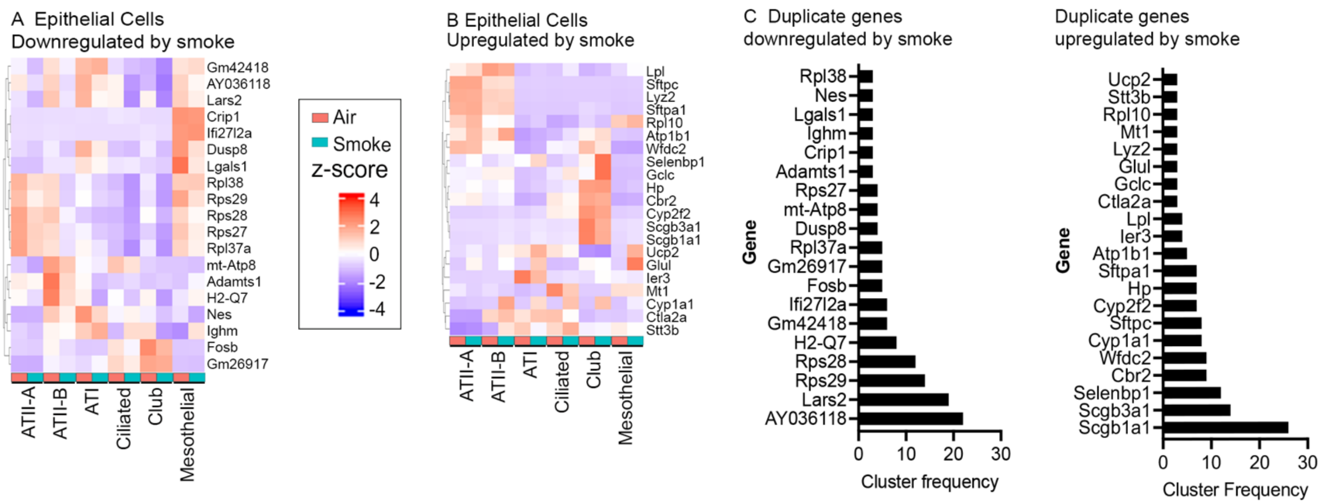

Figure S3. Numerous genes that are differentially expressed across multiple cell clusters.

Heat maps of z-scores showing the top 5 duplicate genes (genes present in >2 cell clusters) that are downregulated and upregulated between air and smoke exposure groups for the epithelial cell clusters **(A)** downregulated by smoke, **(B)** upregulated by smoke, **(C)** Numerous genes are differentially expressed in more than 2 cell clusters (up or downregulated). The cell cluster frequency reflects the number of clusters (includes all cell types) that a gene is differentially expressed in due to smoke exposure, based on an adjusted p-value < 0.05.

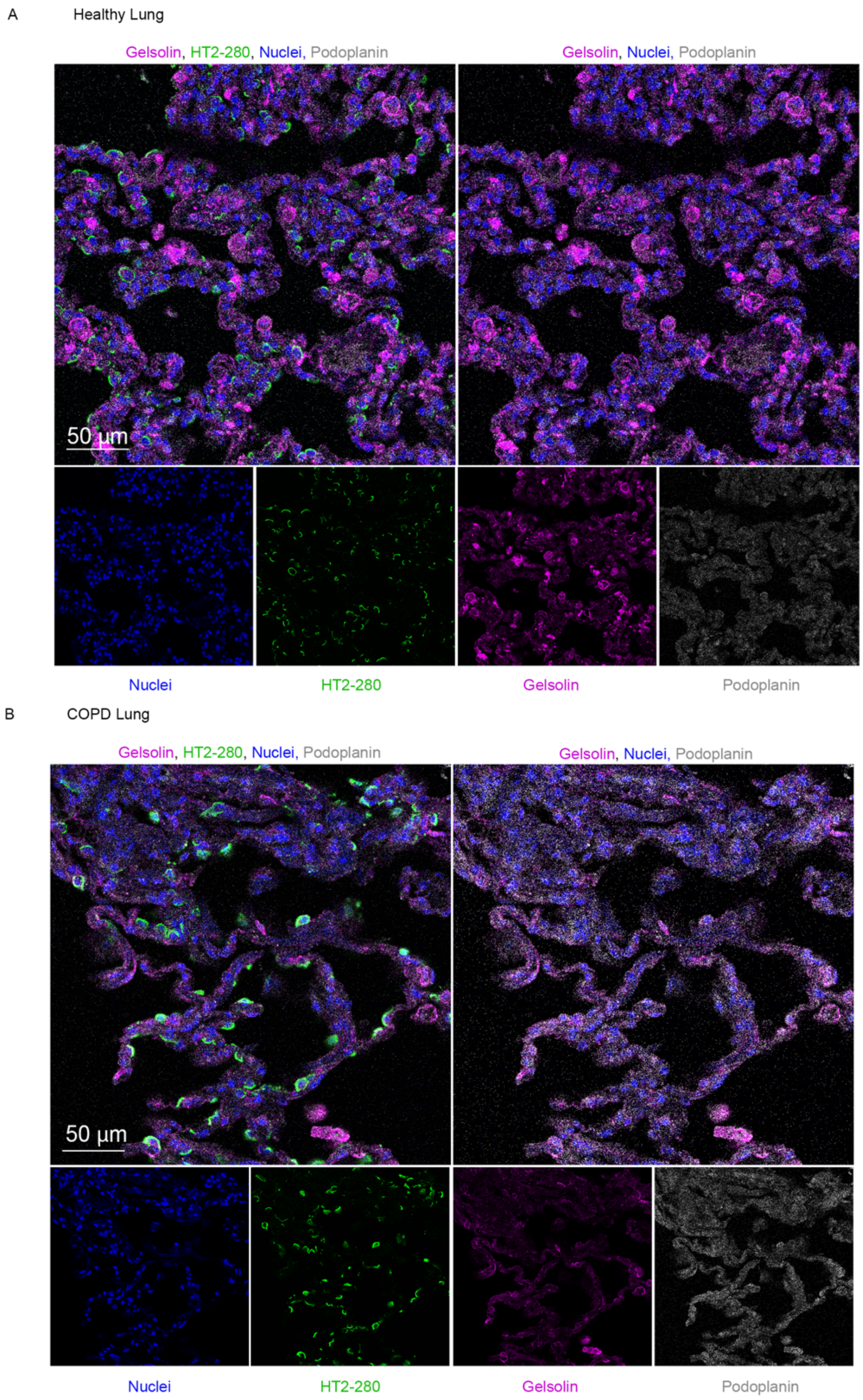

Figure S4. Human alveolar lung tissue stained for GSN, alveolar type 2 cells and alveolar type 1 cells. Human lung tissue sections from control subjects (non-smokers) or patients with COPD were stained by IF and imaged on a confocal microscope n=3-4 subjects per group (8 images per subject). Representative images are shown for (**A**) Human non-smoker lung tissue stained for GSN (magenta), HT2-280 (green), podoplanin (grey) and nuclei with Hoescht stain (blue). Scale bars are 50 µm. (**B**) Human COPD lung tissue stained for GSN (magenta), HT2-280 (green), podoplanin (grey) and nuclei with Hoescht stain (blue). Scale bars are 50 µm.

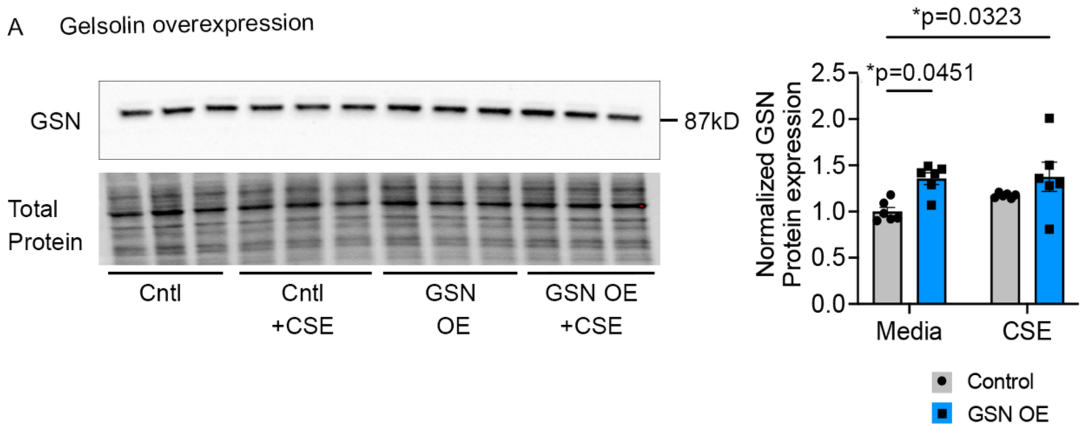

Figure S5. GSN overexpression (OE) in Beas-2b cells. (A) Human bronchial epithelial cells (Beas-2b) were treated with cDNA constructs for overexpression of GSN. Cells were also treated with or without 10% CSE for 24 hours prior to cell lysate collection for western blot analysis of GSN. Western blot band intensities were quantified and normalized to total protein. Data represent normalized GSN band intensity ± standard error mean (SEM). Statistically significant p-values are noted. Statistics by Student's t-test with Mann-Whitney post-test.

Table S1. (separate file)

Number of differentially expressed genes with an adjusted p-value>0.05 by cell type: non-immune and immune cells.

Table S2. (separate file)

Top 5 differentially expressed genes by cell type. Unadjusted and adjusted P values are listed.
