# Supplementary figures and images for: Interpretable machine learning uncovers epithelial transcriptional rewiring and a role for Gelsolin in COPD
Source: JCI Insight. 2024 Nov 8;9(21):e180239. doi: 10.1172/jci.insight.180239 (PMC11601586; doi:10.1172/jci.insight.180239)

Full Blots  
Figure S4  
GSN

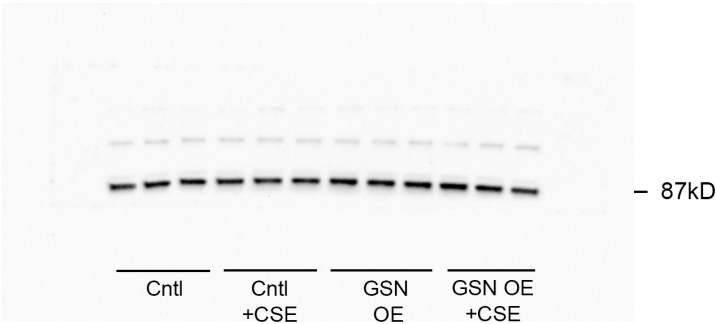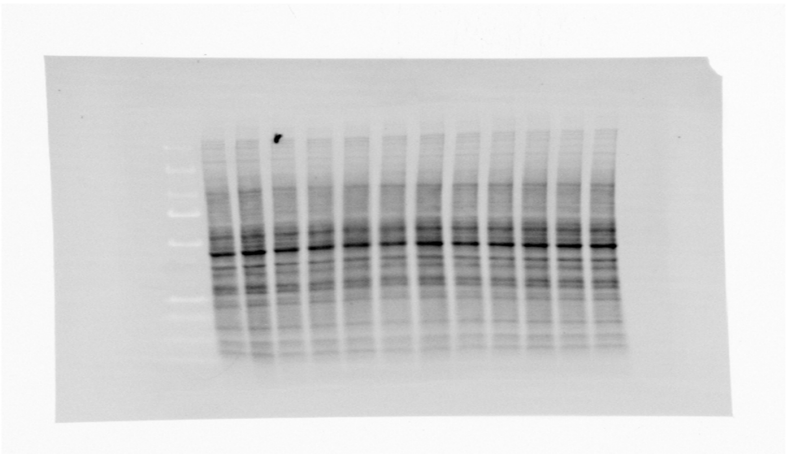

Figure 7  
KRT18

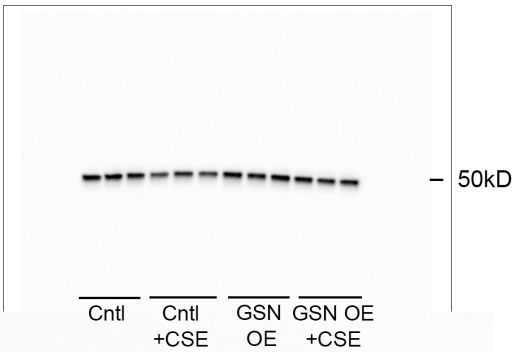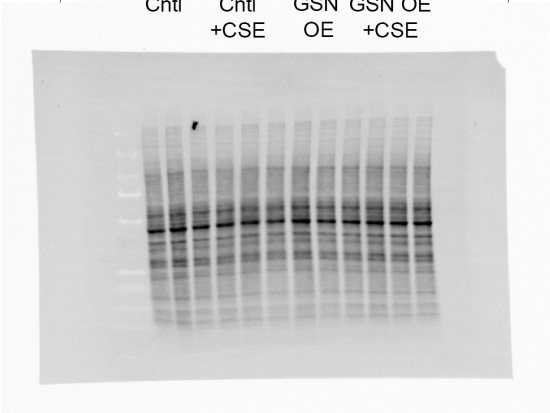

KRT8

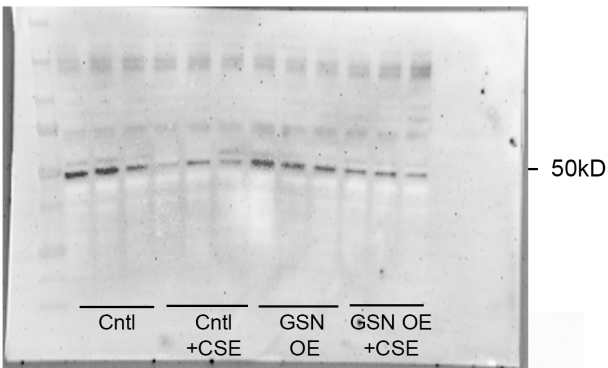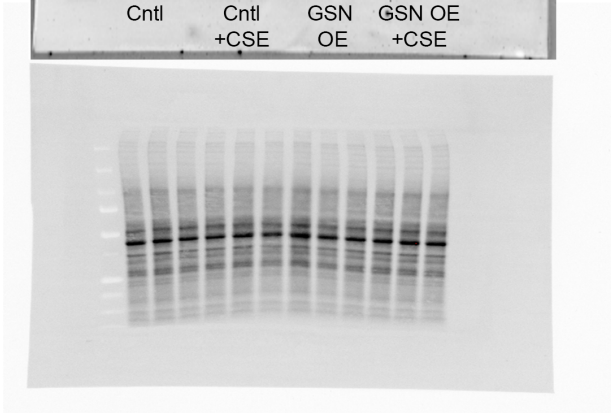

Supplement: Unedited blot and gel images [file jciinsight-9-180239-s256.pdf]
