# Supplementary material for: Interpretable machine learning uncovers epithelial transcriptional rewiring and a role for Gelsolin in COPD
Source: JCI Insight. 2024 Nov 8;9(21):e180239. doi: 10.1172/jci.insight.180239 (PMC11601586; doi:10.1172/jci.insight.180239)
Supplement: Supplemental table 2 [file jciinsight-9-180239-s258.pdf]

Top 5 Downregulated Genes
Epithelial cells

Cell type	Gene	Name	Function	Fold change	P value
ATII-A	Mt1	Metallothionein-1	Anti-oxidant, binds heavy metal ions	0.40713132	1.45E-21
	Lyz1	Lysozyme 1	Antibacterial activity	0.305642467	0.0062
	Areg	Amphiregulin	Epidermal growth factor receptor ligand	0.281697115	2.84E-10
	Scd1	Acyl-CoA desaturase 1	Lipid biosynthesis	0.268094816	1.71E-15
	Elovl1	Fatty acid elongase 1	Catalyzes long-chain fatty acid elongation	0.225450318	2.04E-19
ATII-B	Klf2	Kruppel like factor 2	Activating transcription factor	0.639137534	2.76E-06
	Pltp	Phospholipid transfer protein	Phospholipid transfer protein, cholesterol metabolism	0.510205942	0.000350494
	Atp5md	ATP synthase subunit	Part of the ATP synthase complex V	0.392813434	6.78E-05
	Rpl41	60S ribosomal protein	Protein processing	0.392502369	1.64E-05
	H2-Q6	Histocompatibility complex	Antigen processing and presentation	0.378848958	0.000139867
ATI	Mthfd1	Methylenetetrahydrofolate dehydrogenase 1	Folate metabolism	0.499918519	1.23E-08
	Tomm7	Translocase of outer mitochondrial membrane 7	Mitochondrial outer membrane transporter	0.46559829	4.59E-09
	Ndst1	N-deacetylase/N-sulfotransferase 1	Heparin sulfate processing	0.464103048	5.41E-13
	Ankrd1	Ankyrin repeat domain 1	Transcription factor induced by IL-1 and TNF-alpha	0.462735317	1.02E-08
	Pigp	Phosphatidylinositol Glycan, Class P	Glycolipid anchor biosynthesis	0.46050623	5.37E-09
Ciliated cells	Tuba1b	Tubulin	Cytoskeletal structure	0.600259137	7.92E-10
	Atp5e	ATP synthase subunit	Part of the ATP synthase complex V	0.565884959	6.69E-09
	Ifitm3	Interferon-induced transmembrane protein 3	Interferon induced antiviral proteins, restrict viral entry	0.547680114	0.0002
	Chchd2	Coiled-coil helix protein	Negative regulation of mitochondrial-mediated apoptosis	0.53600446	1.93E-06
	Swi5	Homologous recombination DNA repair protein	Homologous recombination DNA repair protein	0.52309443	4.33E-07
Club cells	Scgb3a2	Secretoglobin member	Secreted lung surfactant protein	0.772420149	1.4E-07
	Lypd2	Ly6/Plaur domain containing protein	Post translational modifications	0.66476357	1.02E-05
	Rpl39	Ribosomal protein	Protein processing	0.662208352	7.45E-13
	Rpl37	Ribosomal protein	Protein processing	0.659056517	6.16E-11
	Pglyrp1	Peptidoglycan recognition protein 1	Pattern receptor with ntimicrobial activity	0.586933449	0.0007
Mesothelial cells	1110004F10Rik	Riken cDNA 1110004F10 gene	Unclear function	0.732569558	0.0014
	Fcf1	Fcf1 rRNA-processing protein	Enable RNA binding	0.677162994	0.0132
	Atp5k	ATP synthase subunit	Part of the ATP synthase complex V	0.630077327	0.0024
	Naxe	NAD(P)HX epimerase	Interacts with apolipoprotein A-I	0.627000316	3.72E-05
	Eno1	Enolase 1	Glycolytic enzyme	0.610102914	0.0086

Top 5 Upregulated Genes
Epithelial cells

Cell type	Gene	Name	Function	Fold change	P value
ATII-A	Prdx6	Peroxiredoxin 6	Glutathione peroxidase and phospholipase activities	-0.428769104	3.68E-99
	Sult1a1	Sulfotransferase family 1a	Catalyze sulfate conjugation of proteins and compounds	-0.372079048	5.65E-52
	Acot1	Acyl-CoA Thioesterase 1	Fatty acid metabolism	-0.370605538	1.06E-52
	Cyp2b10	Cytochrome P450, family 2, subfamily b, polypeptide 10	Cellular detoxification	-0.36977076	3.08E-37
	Ctsh	Cathepsin H	Cysteine protease	-0.349172449	3.16E-56
ATII-B	Adam19	A disintegrin and metalloproteinase 19	Cell surface glycoprotein with proteolytic function	-0.41688692	5.39E-06
	Pon3	Paraoxonase 3	Arylesterase activity; Negative regulation of superoxide anion generation	-0.379552385	1.06E-05
	Npnt	Nephronectin	Enables integrin binding	-0.363876683	4.59E-06
	Mbip	MAP3K12 Binding Inhibitory Protein 1	Protein kinase inhibitor activity; Positive regulation of JNK signaling	-0.36192688	0.0008
	Col4a3	Component of type IV collagen	Collagen structure	-0.337082307	1.42E-06
ATI	Cdkn1a	Cyclin dependent kinase inhibitor 1A ($p21^{Cip1}$)	Cell cycle regulation	-0.679954556	2.03E-10
	Rgcc	Regulator Of Cell Cycle	Cell cycle regulation, induced by p53 due to DNA damage	-0.509374865	2.53E-11
	Sftpb	Surfactant protein B	Surfactant component	-0.502728795	8.45E-05
	Tsc22d3	Also *Gilz*, glucocorticoid-induced leucine zipper	Regulates transcription factor binding	-0.473415263	1.3E-06
	Scgb3a2	Secretoglobin member	Secreted lung surfactant protein	-0.459479134	0.0012
Ciliated cells	Apobec3	Apolipoprotein B editing enzyme catalytic peptide	Deaminase activity, regulates virus replication	-0.668420126	3.58E-14
	Atp8a1	ATP synthase subunit	P-type ATPase; Intracellular transport of cations and ammonia phospholipids	-0.651420926	1.12E-12
	Akr1b8	Aldo-keto reductase family member	oxidoreductase activity	-0.556451482	2.79E-08
	Muc4	Mucin 4	Mucin secreted protein	-0.537323676	1.25E-05
	Resf1	Retroelement silencing factor 1	Regulates repressive	-0.507971155	1.12E-06

			epigenetic modifications		
Club cells	Cyp1b1	Cytochrome p450 superfamily	Monooxygenase involved in the metabolism of various endogenous and drug substrates	-1.895246554	2.56E-61
	Slc7a11	Cystine/glutamate antiporter	Amino acid transporter	-0.934457168	1.33E-12
	Ptgs2	Prostaglandin-endoperoxide synthase 2	Prostaglandin biosynthesis	-0.916573954	3.22E-17
	Nqo1	NAD(P)H dehydrogenase 1	Cellular detoxification	-0.905232409	5.55E-23
	Aox1	Aldehyde oxidase 1	Produces hydrogen peroxide and can catalyze superoxide formation	-0.881969824	2.05E-19
Mesothelial cells	Gstp1	Glutathione S-transferase, pi 1	Regulator of kinase activity	-0.683734533	7.81E-05
	Ryk	Receptor like tyrosine kinase	Enables Wnt activity	-0.613198592	0.0489
	Tax1bp1	Tax 1 binding protein 1	Enables kinase binding, negative regulator of NF-kappaB	-0.590080236	0.0063
	Sorbs3	Sorbin and SH3 domain containing 3	Mediates focal adhesion	-0.482376354	0.0237
	Gcnt2	Glucosaminyl (N-acetyl) transferase 2	Enables acetylglucosaminyl-transferase activity; Regulates epithelial to mesenchymal transition	-0.465935095	0.0450

Top 5 Downregulated Genes
Endothelial and Stromal cells

Cell type	Gene	Name	Function	Fold change	P value
Vasc Endo A	Hlx	Transcription factor	DNA binding and gene expression	0.484686291	2.5E-38
	Nr4a1	Nuclear Receptor Subfamily 4 Group A Member 1	Transcription factor, Can migrate to mitochondria to induce apoptosis	0.475657294	3.23E-40
	Cyp26b1	Cytochrome P450 Family 26 Subfamily B Member 1	Cellular detoxification	0.436129857	1.91E-23
	Dusp1	Dual Specificity Phosphatase 1	Tyrosine and threonine phosphatase	0.407170833	3.4E-60
	Cxcl12	C-X-C Motif Chemokine Ligand 12	Chemoattractant for T-cells and monocytes	0.385155792	2.99E-20
Vasc Endo B	Cldn5	Claudin 5	Integral membrane protein and tight junction component	0.577238948	6.09E-27
	H2-Q6	Histocompatibility complex	Antigen processing and presentation	0.455839452	1.03E-09
	Tspan13	Tetraspanin 13	Cell surface protein regulating cell development, activation, growth and motility	0.444164073	3.9E-13
	H2-Q4	Histocompatibility complex	Antigen processing and presentation	0.438734398	6.46E-12
	H2-Ab1	Histocompatibility complex	Antigen processing and presentation	0.4340207	1.28E-08
Cap Endo A	Tnfsf10	TNF Superfamily Member 10	tumor necrosis factor (TNF) family cytokine; Can trigger MAPK/JNK and apoptosis	0.438422381	1.83E-19
	Gdf15	Growth Differentiation Factor 15	Ligand of the TGF-beta (transforming growth factor-beta) superfamily	0.411155452	0.002129174
	Sep4			0.383196464	1.17E-23
	Cx3cl1	C-X3-C Motif Chemokine Ligand 1	Membrane bound or soluble chemotactic cytokine	0.365789462	1.37E-10
	Cdkn2b	Cyclin dependent kinase inhibitor 2b ($p15^{INK4b}$)	Cell cycle regulation	0.322336407	4.72E-11
Cap Endo B	Atp5md	ATP synthase subunit	Part of the ATP synthase complex V	0.738362635	2.47E-05
	Sparc	Secreted Protein Acidic And Cysteine Rich	extracellular matrix synthesis, cell shape	0.715370031	4.92E-05
	AW112010	AW112010	Transmembrane protein	0.700167608	1.33E-07
	Mien1	Migration And Invasion Enhancer 1		0.644778532	0.000103855
	Uqcc2			0.634618232	3.87E-05
Vcam1+ Endo	Hmcn1			0.458712128	2.95E-09
	Cd74			0.456026457	5.78E-13

	Cnn2			0.409309296	2.14E-07
	H2-Aa	Histocompatibility complex	Antigen processing and presentation	0.398712878	2.91E-09
	Zyx			0.385556885	1.89E-05
Lymph Endo	Apold1			0.573405344	0.000380359
	Ccnd1			0.53507789	0.036516826
	Klf2	Kruppel like factor 2	Activating transcription factor	0.531402908	0.00792549
	Ephb4			0.505128162	0.000565599
	Fos			0.481091052	0.034659278
Interst Fibroblast	Tmem100			0.382027651	5.78E-10
	Olfml3			0.354977166	7.51E-19
	Cnn2			0.352048925	1.6E-16
	Gfra2			0.348011671	4.06E-14
	Mrc2			0.339323294	1.82E-12
Lipofibroblast	Gng11			0.677451048	1.22E-10
	Lrat			0.542103521	1.62E-05
	Gm13889			0.484137664	0.003255783
	Rbp1			0.471267326	7.08E-08
	Mxra8			0.438182661	5.88E-07
Smooth muscle	Lamb1			0.545600939	0.000347922
	Ifitm1			0.541028257	0.008368147
	Cox4i2			0.53646873	0.002491647
	Postn			0.522661197	0.022790319
	Hsd11b1			0.506166183	0.008855104

Top 5 Upregulated Genes
Endothelial and Stromal cells

Cell type	Gene	Name	Function	Fold change	P value
Vasc Endo A	Edn1			-0.684568587	3.84E-78
	Qsox1			-0.526756532	1.39E-82
	Tcn2			-0.521123512	1.45E-124
	Ly6c1			-0.519042507	3.05E-132
	Edc3			-0.495446335	5.87E-70
Vasc Endo B	Sftpb	Surfactant protein B	Surfactant component	-0.6063932	1.6E-13
	Gsn			-0.577633004	3.94E-13
	Scgb3a2	Secretoglobin member	Secreted lung surfactant protein	-0.573729527	9.42E-16
	Prdx6			-0.566724647	1.8E-12
	Cxcl15	C-X-C Motif Chemokine Ligand 15		-0.435123992	1.13E-07
Cap Endo A	Clk3			-0.557361628	7.25E-25
	Ttc39c			-0.42962461	1.26E-12
	Zbtb16			-0.408933011	1.98E-15
	Cdkn1a	Cyclin dependent kinase inhibitor 1A ($p21^{Cip1}$)	Cell cycle regulation	-0.404707731	2.59E-44
	A4galt			-0.386223963	1.08E-14
Cap Endo B	Ablim1			-0.71254561	1.75E-05
	Gm26917			-0.707318907	0.001891462
	Neat1			-0.650013645	0.000103474
	Acap2			-0.644232651	4.09E-05
	Dapk1			-0.629254318	0.005843417
Vcam1+ Endo	Plat			-0.72276401	5.19E-07
	Cyp1b1			-0.677234649	1.59E-09
	Hpgd			-0.508546657	8.13E-14
	Krt80			-0.430697214	3.25E-08
	Gstp1			-0.430494668	0.002077217
Lymph Endo	Thrsp			-0.739572672	0.00020406
	Snhg20			-0.692402794	1.24E-06
	Lcn2			-0.519505386	0.007479082
	Dab2ip			-0.514369139	0.00014217
	Malat1			-0.50330862	0.00193446
Interst Fibroblast	Mt2			-1.029336426	3.66E-23
	Lox			-0.617893708	3.69E-17
	Serpina3n			-0.522910216	1.89E-20
	Timp1			-0.5113842	1.39E-06
	Mpp6			-0.504341294	1.16E-14
Lipofibroblast	Adamts15			-0.544884042	4.31E-06
	Galnt15			-0.508635724	4.93E-08
	Mbd1			-0.499162517	0.000205731
	Prkca			-0.481866589	1.09E-05
	Sftpb	Surfactant protein B	Surfactant component	-0.446497525	0.001367371
Smooth muscle	Sgcd			-0.556209485	7.97E-05
	Mast4			-0.51930464	0.000108103
	Tsc22d3			-0.493692014	0.002243747
	Myom1			-0.479575659	0.010250902
	Slc43a3			-0.478687906	1.85E-05

Myeloid Immune Cells
Top 5 Downregulated Genes

Cell type	Gene	Name	Function	Fold change	P value
Alveolar Mac A	Tuba1b	Tubulin	Cytoskeletal structure	0.273510503	7.59E-30
	Car4	Carbonic anhydrase	Cellular Respiration	0.266829755	2.38E-14
	Marco	Macrophage Receptor with Collagenous Structure	Immune Response	0.260119715	6.6E-09
	Tppp3	Tubulin Polymerization Promoting Protein Family Member 3	Cytoskeletal structure	0.227228229	1.66E-13
	Wfdc21	WAP four-disculfide core domain 21	Immune Response	0.222114192	1.63E-10
Alveolar Mac B	Chil3	Chitinase-like protein 3	Immune Response	0.841703946	7.41E-05
	Psap	Prosaposin	Glycosphingolipid Catabolism	0.621065426	6.47E-07
	Cops9	COP9 signalosome subunit 9	Regulates Ubiquitin conjugation, inflammatory pathway activation	0.550659078	3.17E-06
	Cybb	NADPH oxidase 2	ROS generation	0.537946945	2.99E-05
	Mgat4b	Alpha-1,3-mannosyl-glycoprotein 4-beta-N-acetylglucosaminyltransferase B	Golgi Apparatus regulation	0.519665071	0.000227012
Alveolar Mac C	Sirpa	Signal Regulatory Protein Alpha	Signal Transduction Regulation	0.667843689	0.007782409
	Rtl8a	Retrotransposon Gag-like protein 8A		0.642625459	0.046200673
	Rnf181	Ring Finger Protein 181	E3 Ubiquitin ligase activity	0.627920485	0.020867386
	Brd9	Bromodomain-containing protein 9	Chromatin Remodeling and regulation of transcription	0.624869561	0.009621496
	Capza2	F-actin-capping protein subunit alpha-2	Actin filament cap, regulates growth of actin filaments	0.624291989	0.007097043
Alveolar Mac D	Fabp1	Fatty acid binding protein 1	Transport and metabolism of long chain fatty acids and hydrophobic ligands	1.006609645	0.015460706
	Pmepa1	Prostate Transmembrane Protein, Androgen Induced 1	Androgen receptor and TGF-B pathway suppressor	0.91002451	0.007235437
	Golgb1	Golgin B1	Golgi apparatus regulation	0.814531464	0.002449237
	Snrnp200	Small Nuclear Ribonucleoprotein U5 Subunit 200	Spliceosome pre-RNA splicing	0.776891675	0.001996598
	Commd4	Comm domain containing 4	Modulates cullin-RING E3 ubiquitin ligase complex, down regulate activation of NF-kB	0.717518198	0.015787881

Non-classical Monocyte	Ly6c2	Lymphocyte antigen 6 complex, locus C2	Immune function	0.566657594	4.44E-05
	Fn1	Fibronectin	Tissue repair	0.38386464	0.011737813
	Csf3r	Colony Stimulating Factor 3 Receptor	Immune cell proliferation and differentiation	0.331113942	1.14E-07
	Tmsb10	Thymosin beta-10	Cytoskeleton organization	0.310990566	1.62E-17
	Itgb7	Integrin Beta 7	Cell adhesion and migration	0.296311164	1.02E-05
Interstitial Mac	Lst1	Leukocyte-specific transcript 1	Immune function	0.388206444	3.42E-17
	Zmynd15	Zinc Finger MYND-Type Containing 15	Spermatogenesis	0.374501115	1.48E-05
	Ckb	Creatine Kinase B	Energy homeostasis	0.358335145	2.04E-11
	Mmp12	Matrix Metalloproteinase 12	Extracellular Matrix Breakdown	0.356047474	1.52E-06
	Cd72	Cluster of Differentiation 72	Immune Function	0.344911829	3.84E-10
Cd103+ Dendritic cell	Mgl2	Macrophage galactose N-acetyl-galactosamine specific lectin 2	Carbohydrate binding	0.399991402	0.004112142
	Tes	Testin	Negative regulator of cell growth	0.3566744	0.002814055
	Chd3	Chromodomain-helicase-DNA-binding protein 3	Chromatin remodeling	0.310237147	0.000113079
	Rps21	40s ribosomal protein S21	Ribosomal subunit	0.283593909	7.67E-09
	Pitpnm1	Membrane-associated phosphatidylinositol transfer protein 1	Lipid signal catabolism	0.264364005	0.001859547
Cd209+ Dendritic cell	Bst2	Bone Marrow Stromal Cell Antigen 2	Immune function	0.715648087	8.38E-07
	Cox6a2	Cytochrome c oxidase subunit VIa polypeptide 2	Cellular respiration	0.691645462	1.34E-05
	Klk1	Kallikrein-1	Vasoactive peptide release	0.607558741	8.84E-06
	Cd7	Cluster of Differentiation 7	Immune function	0.598206671	5.46E-08
	Irf8	Interferon Regulatory Factor 8	Immune function	0.546131752	2.78E-05
Eosinophil/Neutrophil	Rnf213	Ring finger protein 213	E3 Ubiquitin ligase	0.457503987	0.012145974
	Sfxn5	Sideroflexin 5	Mitochondrial amino acid transporter	0.422151375	0.024145269
	Id1	Inhibitor of DNA Binding 1	Transcription factor inhibitor	0.409697173	0.016888041
	Itgax	Integrin Subunit Alpha X	Cell adhesion	0.402873186	0.015303801
	Pik3r5	Phosphoinositide-3-Kinase regulatory subunit 5	GPCR signaling	0.394779706	0.049834234
Megakaryocyte	Ptms	Parathymosin	Immune function	1.118726362	9.76E-05

	Ssbp3	Single Stranded DNA Binding Protein 3	Transcriptional regulation of collagen	0.994682954	0.001953286
	Borcs8	BLOC-1 related complex subunit 8	Lysosomal trafficking	0.893616348	0.004929988
	Stx2	Syntaxin 2	Intracellular vesicle trafficking	0.879660036	0.026990647
	Rnf187	Ring finger protein 187	E3 Ubiquitin ligase	0.876534246	0.034091144
Mast cell	Chka	Choline Kinase Alpha	Phospholipid biosynthesis	0.816859833	0.005449014
	Tra2b	Transformer 2 Beta Homolog	mRNA splicing	0.726840723	0.002448372
	Ccdc88c	Coiled-coil domain containing 88c	Wnt pathway regulation	0.685560841	0.004449019
	Chst15	Carbohydrate Sulfotransferase 15	B cell activation regulation	0.674972155	0.025638385
	Siglecf	Sialic acid binding lg-like lectin F	Endocytosis regulation	0.640800034	0.006374497

Myeloid Immune Cells
Top 5 Upregulated Genes

Cell type	Gene	Name	Function	Fold change	P value
Alveolar Mac A	Ap1s2	Adaptor Related Protein Complex 1 Subunit Sigma 2	Golgi apparatus function	-0.247351657	7.8E-26
	S100a6	S100 Calcium Binding Protein A6	Calcium Sensor, cell proliferation	-0.239620467	2.42E-06
	Srgn	Serglycin	Granule-mediated apoptosis	-0.23572098	2.02E-18
	Cdc42ep3	CDC42 Effector Protein 3	Cytoskeletal remodeling	-0.2157595	1.59E-19
	Net1	Neuroepithelial Cell Transforming 1	DNA damage repair	-0.21176935	6.41E-18
Alveolar Mac B	Nupr1	Nuclear Protein 1, Transcriptional Regulator	Transcriptional regulator	-0.678774743	0.002511343
	Scgb3a2	Secretoglobin member	Secreted lung surfactant protein	-0.661904841	0.004029565
	Mgp	Matrix Gla Protein	Inhibitor of bone formation	-0.639635174	0.003717641
	Abi1	Abl Interactor 1	Cell growth regulation, cytoskeleton regulation	-0.559119198	0.002920906
	Trafd1	TRAF-type Zinc Finder Domain Containing 1	Immune activation regulator	-0.545997903	0.015579618
Alveolar Mac C	Xist	X-inactive specific transcript	X-inactivation	-1.063004134	0.021168034
	Pag1	Phosphoprotein Membrane Anchor with Glycosphingolipid Microdomains 1	Negative regulator of T cell activation	-1.042268013	0.000756524
	Kcnq1ot1	KCNQ1 Opposite Strand/Antisense Transcript 1	Epigenetic regulation	-1.013331419	0.000381053
	Lyve1	Lymphatic Vessel Endothelial Hyaluronan Receptor 1	Cell growth	-0.922275813	0.000489562
	Hpgd	15-Hydroxyprostaglandin Dehydrogenase	Prostaglandin metabolism	-0.918559427	0.000524535
Alveolar Mac D	Arhgef2	Rho/Rac Guanine Nucleotide Exchange Factor 2	Rho-GTPase activation	-0.854550997	0.013721951
	Prex1	Phosphatidylinositol-3,4,5-Triphosphate Dependent Rac Exchange Factor 1	Rac protein activation	-0.825724038	0.002443832
	Inpp5k	Inositol Polyphosphate-5-Phosphatase K	Actin cytoskeleton regulation	-0.715746149	0.002382849
	Dexi			-0.696589636	0.002762447
	Rnf216	Ring finger protein 216	E3 Ubiquitin ligase	-0.692064725	0.00960085

Non-classical Monocyte	Fabp4	Fatty acid binding protein 4	Lipid transport	-0.623440342	9.65E-17
	Pltp	Phospholipid Transfer Protein	Lipid transport	-0.556463267	2.82E-18
	Serpinb6a	Serine peptidase inhibitor, clade B, member 6a	Response to osmotic stress	-0.524693621	4.99E-16
	Acp5	Acid phosphatase 5, tartrate resistant	Osteopontin regulation	-0.516319231	6.37E-15
	Fcgr4	FC receptor, IgG, low affinity IV	Immune function	-0.506448382	5.48E-14
Interstitial Mac	Cxcl13	C-X-C Motif Chemokine Ligand 13	B cell chemotaxis	-1.100464656	1.48E-10
	Ms4a4a	Membrane spanning 4-domains A4A	Signal transduction	-0.903397803	2.56E-36
	Ccl6	Chemokine ligand 6	Chemokine	-0.534510396	1.4E-14
	Ccl7	C-C motif chemokine ligand 7	Chemokine	-0.521515661	5.28E-05
	C5ar1	Complement C5a Receptor 1	Immune function	-0.492373901	1.4E-18
Cd103+ Dendritic cell	Apol7c	Apolipoprotein L 7c	Lipid transport	-0.754272623	0.035145208
	Slco3a1	Solute Carrier Organic Anion Transporter Family Member 3A1	Transporter activity	-0.473633697	3.92E-08
	Asb2	Ankyrin Repeat and SOCS Box Containing 2	E3 Ubiquitin ligase	-0.453178169	0.000165031
	Gm43305	Predicted gene 43305	Unknown	-0.439558832	0.019485648
	Ddit4	DNA damage inducible transcript 4	Cell growth regulation	-0.407292262	3.69E-06
Cd209+ Dendritic cell	Malat1	Metastasis Associated Lung Adenocarcinoma Transcript 1	Transcriptional regulation	-0.490971543	0.010371074
	Gstm1	Glutathione S-transferase mu 1	Cellular detoxification	-0.481921437	0.00340437
	Spint1	Serine peptidase inhibitor, Kunitz type 1	HGF signaling regulation	-0.47404391	1.66E-07
	Tgfbi	Transforming growth factor beta induced	Cell adhesion and migration	-0.402381888	0.002260815
	Pid1	Phosphotyrosine interaction domain containing 1	Cell proliferation	-0.380992971	0.001315943
Eosinophil/Neutrophil	Cd14	CD14 molecule	Immune function	-0.58409418	0.00012254
	Cd74	CD74 molecule	Immune function	-0.524856478	0.001155688
	Cxcl2	C-X-C Motif Chemokine Ligand 2	Chemokine	-0.475819993	0.003743345
	Vav3	Vav Guanine Nucleotide Exchange Factor 3	Cytoskeletal regulation	-0.473291282	0.000568903
	Serinc3	Serine Incorporator 3	Immune function	-0.469411372	3.77E-06
Megakaryocyte	Hspa8	Heat shock protein family A member 8	Molecular chaperone	-1.235100135	0.001891233
	Hcfc1r1	Host Cell Factor C1 Regulator 1	Transcription factor regulator	-1.128537731	0.011536195
	Fkbp1a	FKBP prolyl isomerase 1A	Immunoregulation, protein folding	-1.053619365	0.008227779
	Cul1	Cullin 1	E3 Ubiquitin ligase	-1.050701155	0.010747036

	H2-K1	Histocompatibility complex	Antigen processing and presentation	-1.022745153	0.001052201
Mast cell	Mcpt8	Mast cell protease 8	Serine-type endopeptidase	-1.551694146	0.000520763
	Slpi	Secretory leukocyte peptidase inhibitor	Protection against protease, immune function	-1.354286929	0.000186028
	Adam8	ADAM Metallopeptidase Domain 8	Cell adhesion	-0.79153508	0.000391682
	Tnik	TRAF2 and NCK interacting kinase	Wnt signaling activator	-0.738180461	0.002244456
	Mbd6	Methyl-CpG Binding Domain Protein 6	Chromatin binding activity	-0.706575396	0.000466679

Lymphoid Immune Cells
Top 5 Downregulated Genes

Cell type	Gene	Name	Function	Fold change	P value
B cell - A	Igkc	Immunoglobulin Kappa Constant	Immune function	0.467604047	1.87E-46
	Ighd	Immunoglobulin Heavy Constant Delta	Immune function	0.291093595	4.72E-22
	Cd22	Siglec2	Immune function	0.261765481	1.04E-15
	Flna	Filamin A	Actin cytoskeletal remodeling	0.255065469	1.77E-13
	Gm47283	Gm47283	unknown	0.252883654	9.84E-14
B cell - B	Scn7a	Sodium Voltage-Gated Channel Alpha Subunit 7	Sodium channel	0.993116868	0.016553228
	Polr2m	RNA Polymerase II Subunit M	Negative regulator of transcription	0.846981675	0.005870444
	Tie1	Tyrosine Kinase with Immunoglobulin like and EGF like Domains 1	Angiogenesis	0.812093651	0.005331791
	Suz12	SUZ12 Polycomb Repressive Complex 2 Subunit	Epigenetic repression	0.794867372	0.012209032
	Snrk	SNF Related Kinase	Hematopoietic proliferation or differentiation	0.790461883	0.024159005
Cd4+ T cell	Abca2	ATP binding cassette subfamily A member 2	Protein trafficking	0.375254793	0.000908593
	Bcl11b	BAF chromatin remodeling complex subunit BCL11B	Transcriptional repressor	0.335661234	0.001900776
	Sema6d	Semaphorin 6D	Neuronal axon pathfinding	0.322284059	0.004301313
	Stab2	Stabilin 2	Angiogenesis, lymphocyte homing, cell adhesion, receptor scavenging	0.319086525	0.005237718
	Tcrg-C1	T cell receptor gamma constant 1	Immune function	0.309891225	0.004545668
Cd8+ T cell - A	Ccl5	C-C Motif Chemokine Ligand 5	Immune function	0.87756628	0.033043133
	S100a4	S100 Calcium Binding Protein A4	Cell cycle progression and differentiation	0.415150896	1.49E-05

	Maf	MAF BZIP Transcription Factor	Transcriptional activator or repressor	0.395658835	3.46E-11
	Ikzf2	IKAROS Family Zinc Finger 2	Transcription factor for lymphocyte development	0.338487654	1.02E-06
	Izumo1r	IZUMO1 Receptor, JUNO	Folic acid pathway	0.330145855	1.27E-07
Cd8+ T cell - B	Trbv31	T cell receptor beta, variable 31	Cell signaling	0.595683922	6E-10
	Trbc2	T cell receptor beta constant 2	Immune function	0.353063687	2.22E-06
	Tnrc6b	Trinucleotide repeat containing adaptor 6B	miRNA and siRNA Transcriptional repression	0.267873995	9.1E-05
	Sh2d1a	SH2 Domain Containing 1A	Immune function	0.241900081	0.002757828
	Atp2b1	ATP synthase subunit	Part of the ATP synthase complex V	0.240071281	0.021916721
Cd8+ T cell – C	S100a6	S100 calcium binding protein A6	Cell cycle progression and differentiation	1.844542529	0.001647671
	Ckb	Creatine Kinase B	Energy homeostasis	1.397853489	0.000215867
	Cxcr6	C-X-C Motif Chemokine Receptor 6	T cell migration	1.223055594	0.0002368
	Ramp1	Receptor Activity Modifying Protein 1	Calcitonin receptor like receptor transporter	1.038291981	0.015121942
	Lmo4	LIM Domain Only 4	Transcriptional regulator	0.998889804	0.019226917
NK cell	Klra7	Killer cell lectin-like receptor 7	MHC I receptor	0.467341877	2.25E-05
	Thy1	Thy-1 Cell Surface Antigen	Cell adhesion and cell communication	0.420344759	0.006089684
	Rpl39	Ribosomal protein	Protein processing	0.381430893	9.93E-11
	Kcnq1ot1	KCNQ1 opposite strand/antisense transcript 1	Epigenetic regulation	0.356339169	0.000128643
	Slc12a7	Solute Carrier Family 12 Member 7	Potassium-chloride transport	0.349869106	6.7E-05
Cd4+ or Cd8+ T cell	Tnfrsf4	TNF Receptor Superfamily Member 4	NF-kappaB activator	0.55905306	0.002003274
	Stat1	Signal Transducer and Activator of Transcription 1	Transcriptional activator	0.463842225	0.004659337
	Cdk6	Cyclin Dependent Kinase 6	Cell cycle control and differentiation	0.398818276	7.65E-05

	Trbc1	T Cell Receptor Beta Constant 1	Immune function	0.377617396	1.18E-05
	Ms4a4b	Membrane Spanning 4-Domains A4A	Cell cycle modulation	0.368183467	0.000498317

Lymphoid Immune Cells
Top 5 upregulated genes

Cell type	Gene	Name	Function	Fold change	P value
B cell - A	March1	Membrane-Associated Ring Finger (C3HC4) 1, E3 Ubiquitin Protein Ligase	Vesicular transport	-0.276485558	1.95E-19
	Rps15	Ribosomal Protein S15	Ribosomal subunit	-0.227016479	5.14E-25
	Zfp36l2	ZFP36 Ring finger Protein Like 2	Growth Factor Response	-0.226791376	9.82E-16
	Sftpb	Surfactant protein B	Surfactant component	-0.208002127	2.83E-10
	Tsc22d3	TSC22 Domain Family Member 3	Anti-inflammatory	-0.199454324	1.1E-15
B cell - B	Trim33	Tripartite Motif Containing 33	E3 Ubiquitin Ligase	-0.773340525	0.001380153
	Leprot	Leptin Receptor Overlapping Transcript	Growth hormone receptor regulation	-0.757998341	0.002550324
	Stat4	Signal Transducer and Activator of Transcription 4	Immune function	-0.747090443	0.011800575
	Adrb2	Adrenoceptor Beta 2	G protein-coupled receptor	-0.723658249	0.003271734
	Bcap31	B Cell Receptor Associated Protein 31	Chaperone Protein	-0.721467114	0.011976898
Cd4+ T cell	Calca	Calcitonin Related Polypeptide Alpha	Calcium Regulation	-0.850865203	3.71E-07
	Pard3	Par-3 Family Cell Polarity Regulator	Cell Polarization	-0.462064528	1.54E-08
	Prnp	Prion Protein	Unknown	-0.397165751	3.78E-05
	Gsn	Gelsolin	Actin Cytoskeletal Regulation	-0.369490406	0.002730772
	Ccr8	C-C Motif Chemokine Receptor 8	Immune function	-0.35277029	5.26E-06
Cd8+ T cell - A	Rpl5	Ribosomal protein	Protein processing	-0.335674371	3.15E-35
	Gm21887	Gm21887	Unknown	-0.278877586	6.55E-11
	Txnip	Thioredoxin Interacting Protein	Antioxidant inhibitor	-0.251908867	1.7E-15
	Gm20400	Gm20400	Unknown	-0.238170747	1.43E-07
	Rack1	Receptor for Activated C Kinase 1	Cell signaling regulator, Ribosomal regulator	-0.223202095	9.67E-22
Cd8+ T cell - B	Gzmb	Granzyme B	Induction of apoptosis	-0.299115217	0.013548765
	Itgb1	Integrin Subunit Beta 1	Cell adhesion	-0.292971974	1.21E-06
	Gm2682	Gm2682	Unknown	-0.283478695	7.13E-08
	Cd8b1	CD8b Molecule	Immune function	-0.266462896	0.000609489

	Il7r	Interleukin 7 Receptor	Immune function	-0.24816317	0.009180888
Cd8+ T cell - C	Edn1	Endothelin 1	Vasoconstrictor	-1.214566821	0.021698065
	Ccr7	C-C Motif Chemokine Receptor 7	T cell migration	-0.923751533	0.003818504
	Cd28	CD28 Molecule	T cell proliferation and survival	-0.851640447	0.01007102
	Sox7	SRY-Box Transcription Factor 7	Embryonic Development Regulation	-0.851290095	0.001397878
	Ly6c2	Lymphocyte antigen 6 complex, locus C2	Immune Function	-0.786777621	0.010194773
NK cell	Gm43305	Gm43305	Unknown	-0.492185199	0.02269418
	S1pr5	Sphingosine-1-Phosphate receptor 5	G Protein-coupled receptor, cell signaling	-0.384887294	2.37E-07
	Arhgap15	Rho GTPase Activating Protein 15	Actin cytoskeletal modulation	-0.377317284	0.020105534
	Sap30l	Sap30 Like	Histone deacetylase recruitment	-0.357508402	0.001913869
	Prkar1a	Protein Kinase CAMP-Dependent Type I Regulatory Subunit	Lipid and glucose metabolism	-0.34778608	0.000337732
Cd4+ or Cd8+ T cell	Asb2	Ankyrin Repeat and SOCS Box Containing 2	E3 Ubiquitin Ligase	-0.342980516	0.000120752
	Large1	LARGE Xylosyl-And Glucuronyltransferase 1	Glycoprotein synthesis	-0.333599113	0.000351978
	Slc9a9	Solute Carrier Family 9 Member A9	Organelle ion homeostasis	-0.308725666	1.92E-05
	Ddit4	DNA Damage Inducible Transcript 4	Cell growth, proliferation and survival regulation	-0.239917936	2.76E-06
	Cog1	Component of Oligomeric Golgi Complex 1	Golgi structure	-0.233543881	3.31E-07
